# Supplementary material for: Plant community stability is associated with a decoupling of prokaryote and fungal soil networks
Source: Nat Commun. 2023 Jun 22;14:3736. doi: 10.1038/s41467-023-39464-8 (PMC10287681; doi:10.1038/s41467-023-39464-8)
Supplement: Supplementary file 1 — Supplementary Information [file 41467_2023_39464_MOESM1_ESM.pdf]

Plant community stability is associated with a decoupling of prokaryote and fungal soil networks

Dina in 't Zandt[1], Zuzana Kolaříková[1], Tomáš Cajthaml[2,3] and Zuzana Münzbergová[1,4]

[1]Institute of Botany, Czech Academy of Sciences, 252 43 Průhonice, Czech Republic

[2]Institute for Environmental Studies, Faculty of Science, Charles University, Praha 2, Czech Republic

[3]Institute of Microbiology, Czech Academy of Sciences, Vídeňská 1083, Prague CZ-14220, Czech Republic

[4]Department of Botany, Faculty of Science, Charles University, Praha 2, Czech Republic

Corresponding author: Dina in 't Zandt, e-mail: dina.intzandt@ibot.cas.cz

Supplementary Figures

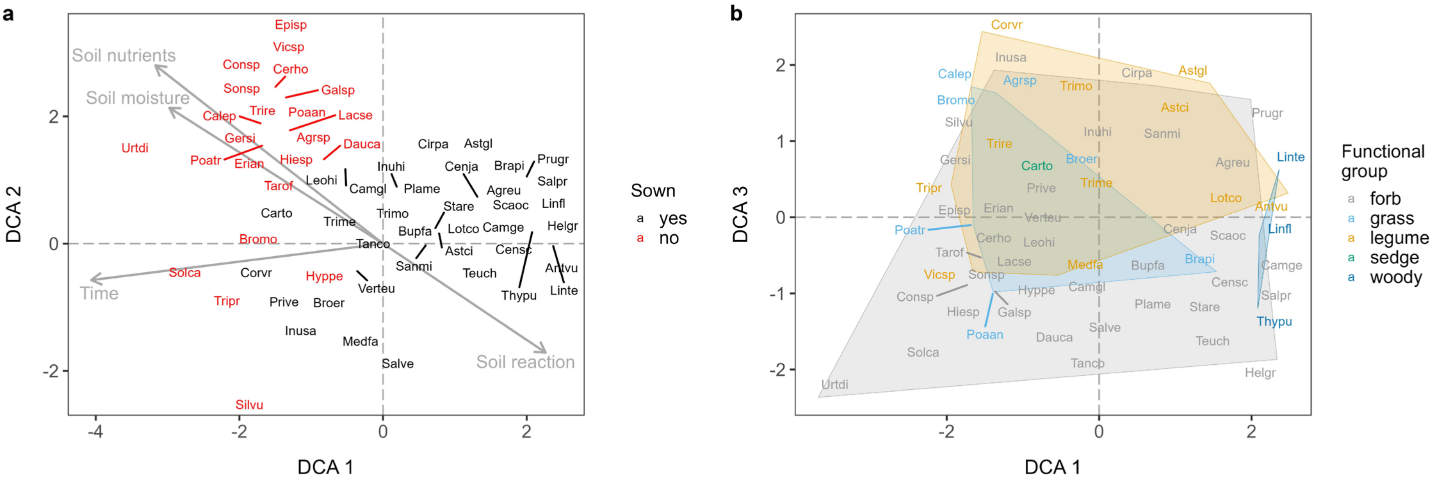

Supplementary Figure 1. **Plant community compositional development.** Plant species position on detrended correspondence analysis (DCA) axes (**a**) 1 and 2, and (**b**) 1 and 3 (n = 59 plant species; both soil origins included). In a, arrows show a passive overlay of significant ($p < 0.05$) plant factors with Time indicating the average time period (year between 2007 and 2019) that plant species were present in the communities, and Soil nutrients, Soil moisture and Soil reaction referring to plant species ecological optima based on Czech Ellenberg indicators. Plant species abbreviations shown in black are sown species, while species in red invaded the communities. In b, hulls indicate the section of the DCA axes where plant species of five functional groups occur and shows that DCA 3 separates communities based on legume cover. For plant species abbreviations, see Supplementary Table 9. For statistics, see Supplementary Data 1. Source data are provided as a Source Data file.

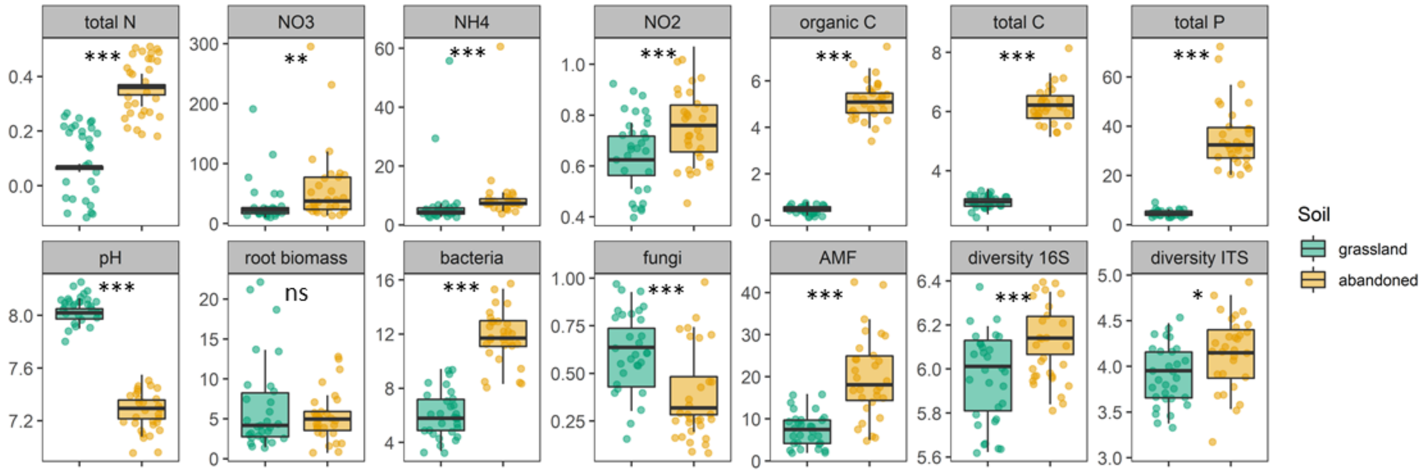

Supplementary Figure 2. **Soil chemical and microbial properties after the 13th growing season**. Plant communities were grown on natural grassland (green) and abandoned arable (yellow). Total N, total C and organic C are given in percentage; NO_3^-, NH_4^+, NO_2^-, K and P, as well as bacterial, fungal and AMF biomass are given in mg · kg^{-1} dry soil; root biomass is given in gram, and for 16S and ITS diversity the Shannon diversity index is shown. Averages ± SE (n = 30), asterisks indicate significant differences between natural grassland and abandoned arable soil. Results of linear mixed effect models with sowing density as a random effect and type III Wald chi-square tests are presented. Significance codes: *** p < 0.001; ** p <0.01; * p<0.05; ns, not significant, p>0.05. For exact statistical values, see Supplementary Data 1. Source data are provided as a Source Data file.

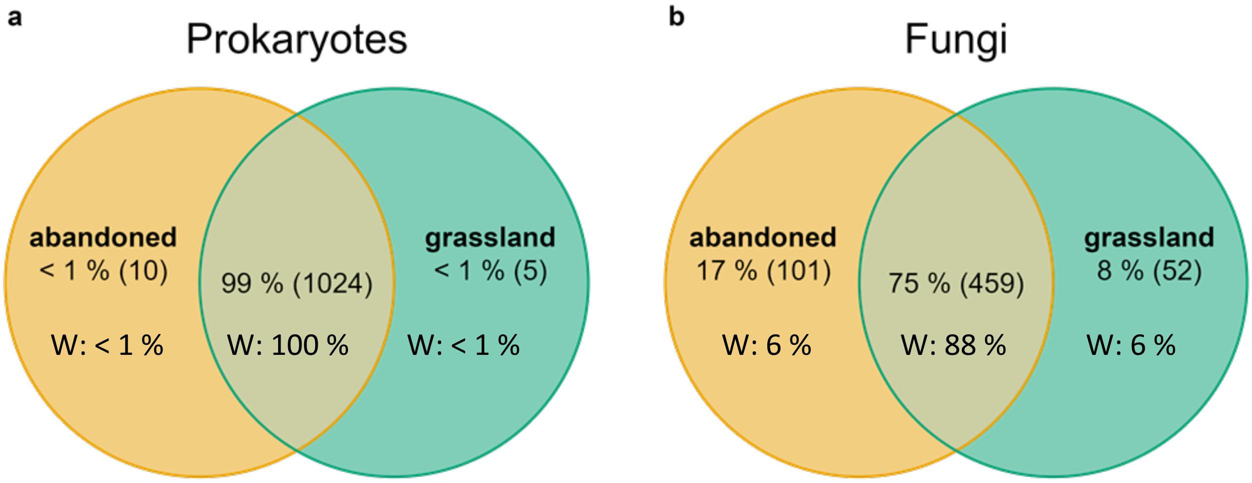

Supplementary Figure 3. **Venn diagram prokaryote and fungal overlap.** (**a**) Prokaryote and (**b**) fungal OTUs between natural grassland (green) and abandoned arable soil (yellow). In the top row, the number of OTUs in each compartment is indicated in percentages and, in brackets, as the total number. The bottom row shows the percentage of OTUs in each compartment weighted by the OTUs relative abundance (*n* = 30 per soil origin).

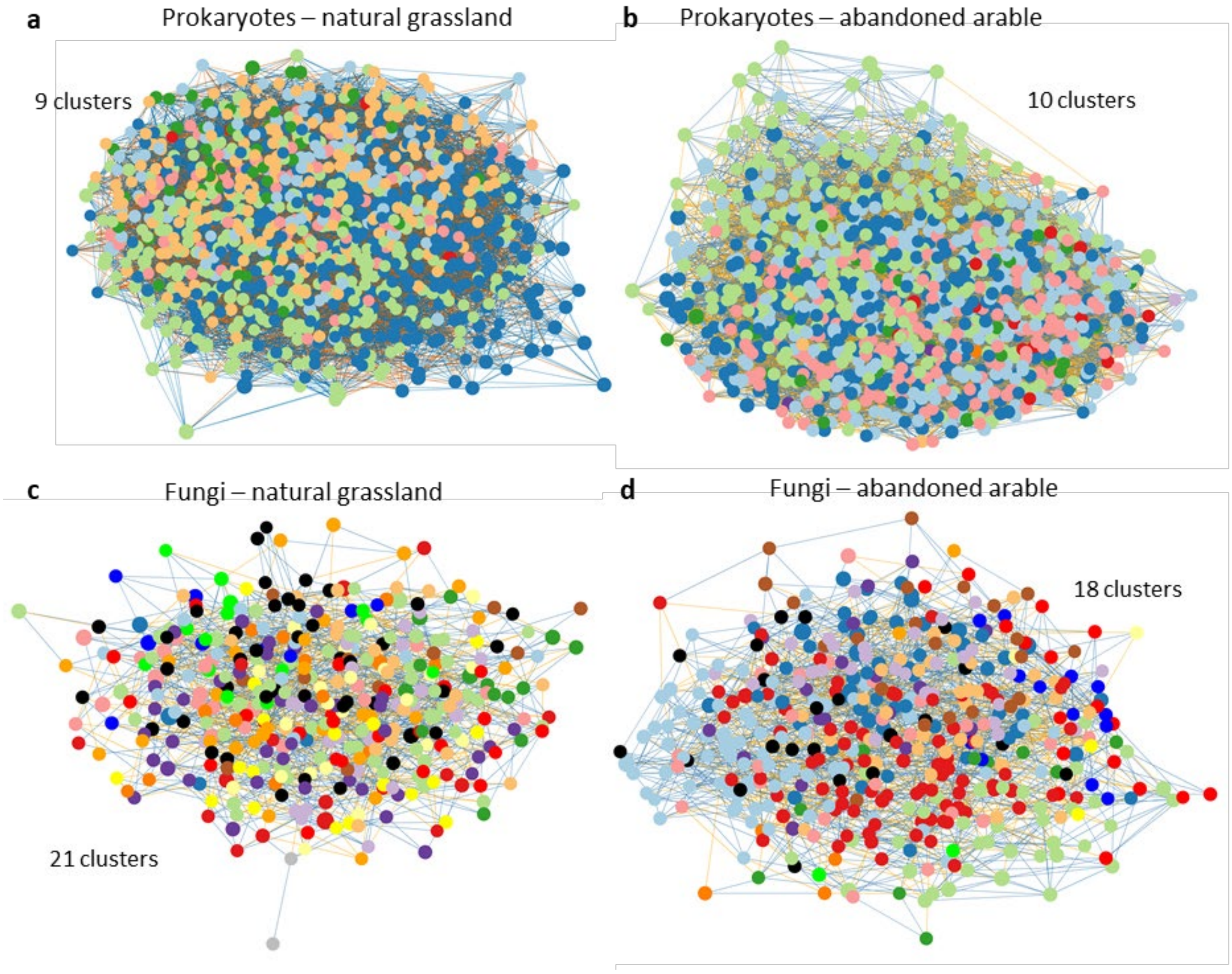

Supplementary Figure 4. **Soil microbial co-occurrence networks.** Prokaryote networks in (**a**) natural grassland soil and (**b**) in abandoned arable soil, and of fungal networks in (**c**) natural grassland soil and (**d**) abandoned arable soil. Each dot represents one OTU. Different colours indicate that OTUs belonged to different clusters within the network (*n* =30 per soil origin).

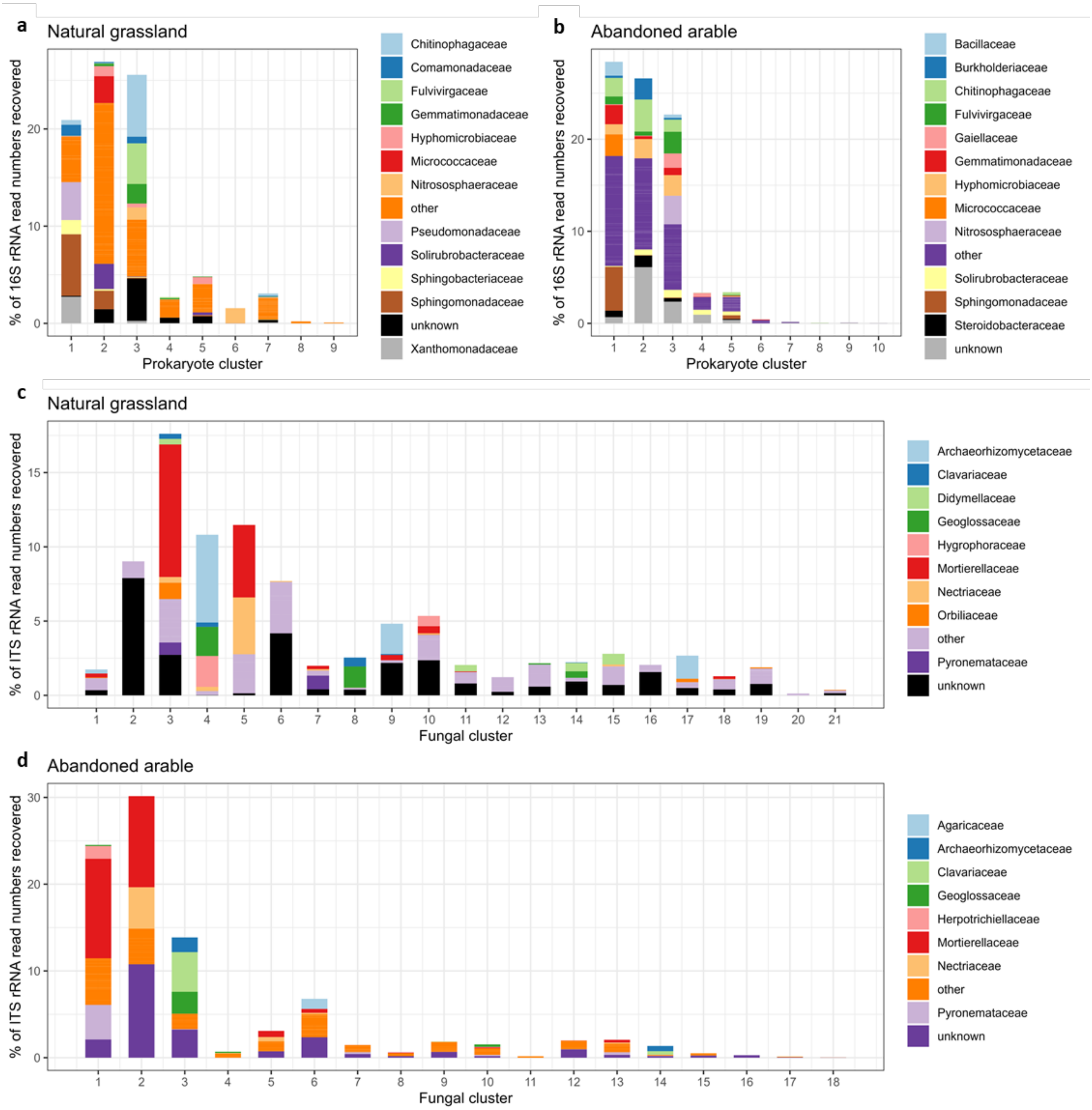

Supplementary Figure 5. **Taxonomic characterisation of prokaryote and fungal network clusters.** Average percentage of 16S and ITS rRNA read numbers recovered per family level in prokaryote clusters in (**a**) natural grassland and (**b**) abandoned arable soil, and fungal clusters in (**c**) natural grassland and (**d**) abandoned arable soil. Microbial clusters were obtained from co-occurrence networks (Fig. 3; Supplementary Fig. 4). For 16S, families < 2% relative abundances are grouped in other, for ITS, families <1.5% relative abundances are grouped in other. Source data are provided as a Source Data file.

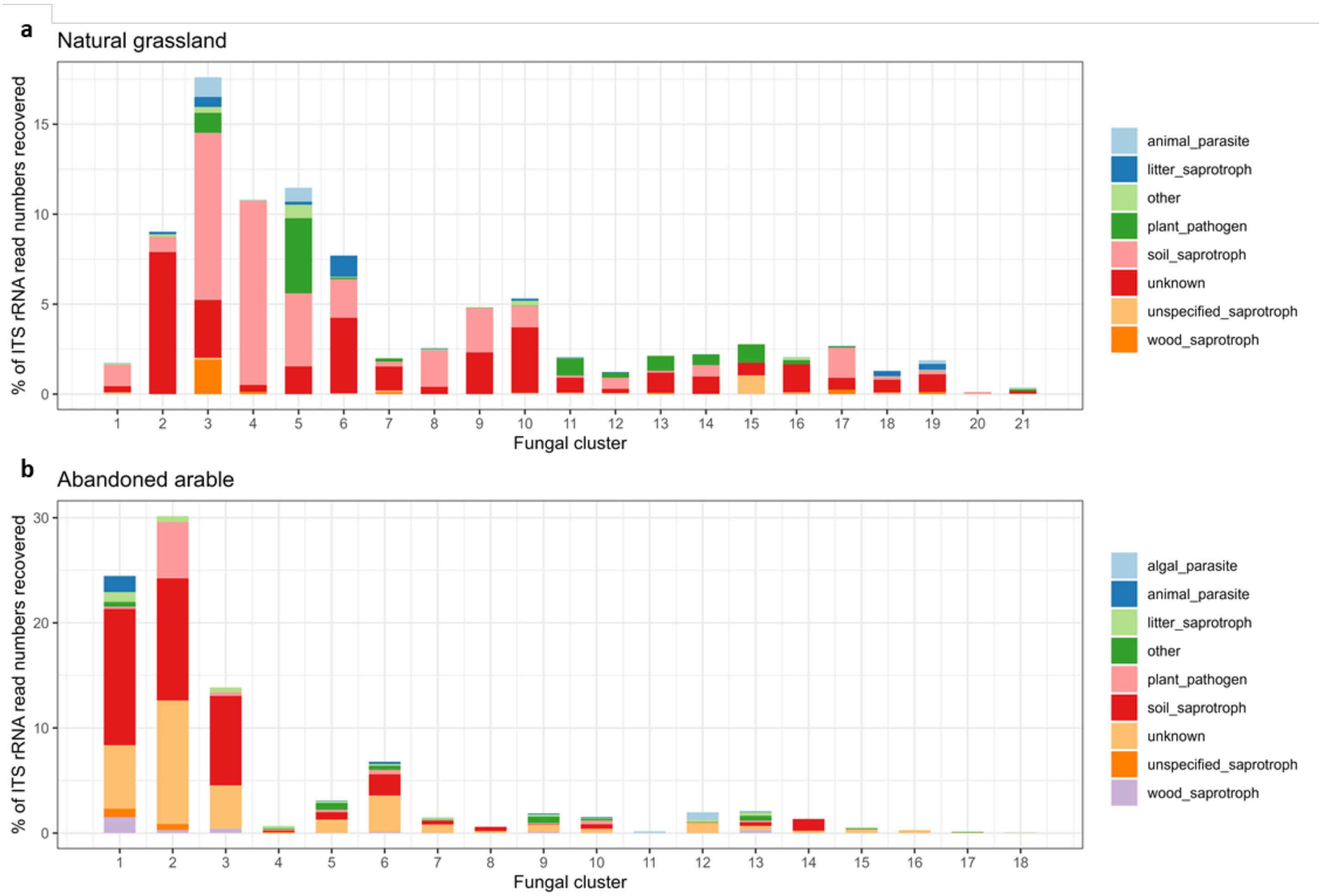

Supplementary Figure 6. **Putative fungal traits of fungal network clusters.** Average percentage of ITS rRNA read numbers recovered per putative fungal trait over similarly responding fungal clusters obtained from co-occurrence networks in (**a**) natural grassland and (**b**) abandoned arable soil. Putative fungal traits < 1% relative abundances are grouped in other. Fungal traits were obtained from the FungalTraits database (Põlme, S. et al. 2020. Fungal Divers 105). Source data are provided as a Source Data file.

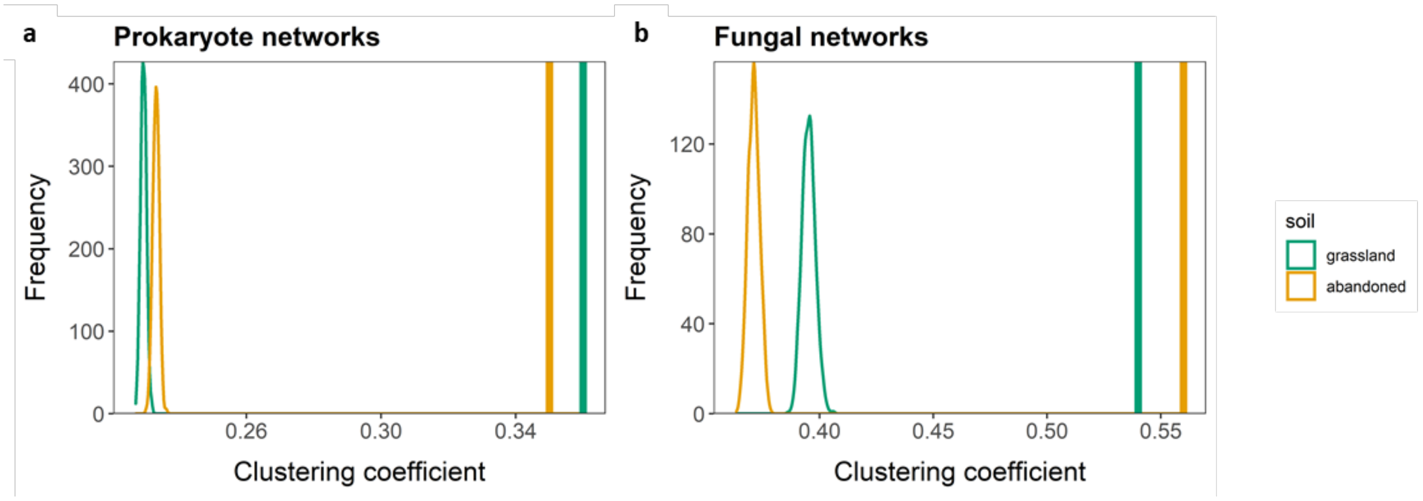

Supplementary Figure 7. **Null-modelling of network clusters.** Frequency distribution of clustering coefficients (modularity) in random networks of (**a**) prokaryote and (**b**) fungal co-occurrence networks in natural grassland (green) and abandoned arable soil (yellow). Vertical lines indicate the clustering coefficients of the real microbial networks. Random networks were created by rewiring the edges of the original networks while preserving the original networks degree distribution (1000 iterations). The clustering coefficients of the original networks never occurred in the rewired networks. Source data are provided as a Source Data file.

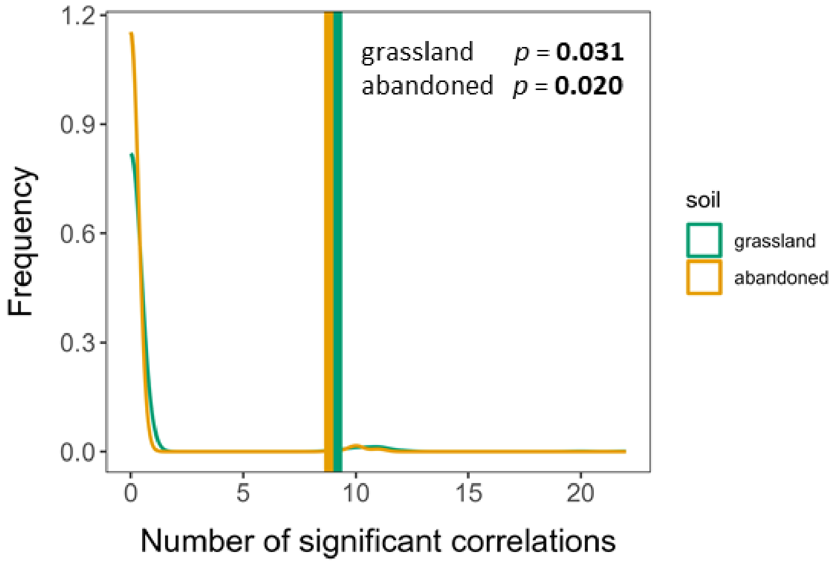

Supplementary Figure 8. **Null-modelling of prokaryote and fungal network coupling.** Frequency distribution of the number of significant correlations between each prokaryote and fungal cluster in random co-occurrence networks in natural grassland (green) and abandoned arable soil (yellow). Vertical lines indicate the number of significant correlations in the real microbial networks (9 for both soils). Random networks were created by rewiring the edges of the original networks while preserving the original network degree distribution (1000 iterations). *P*-values were calculated as the proportion of randomised number of significant correlations smaller than in the original networks. Source data are provided as a Source Data file.

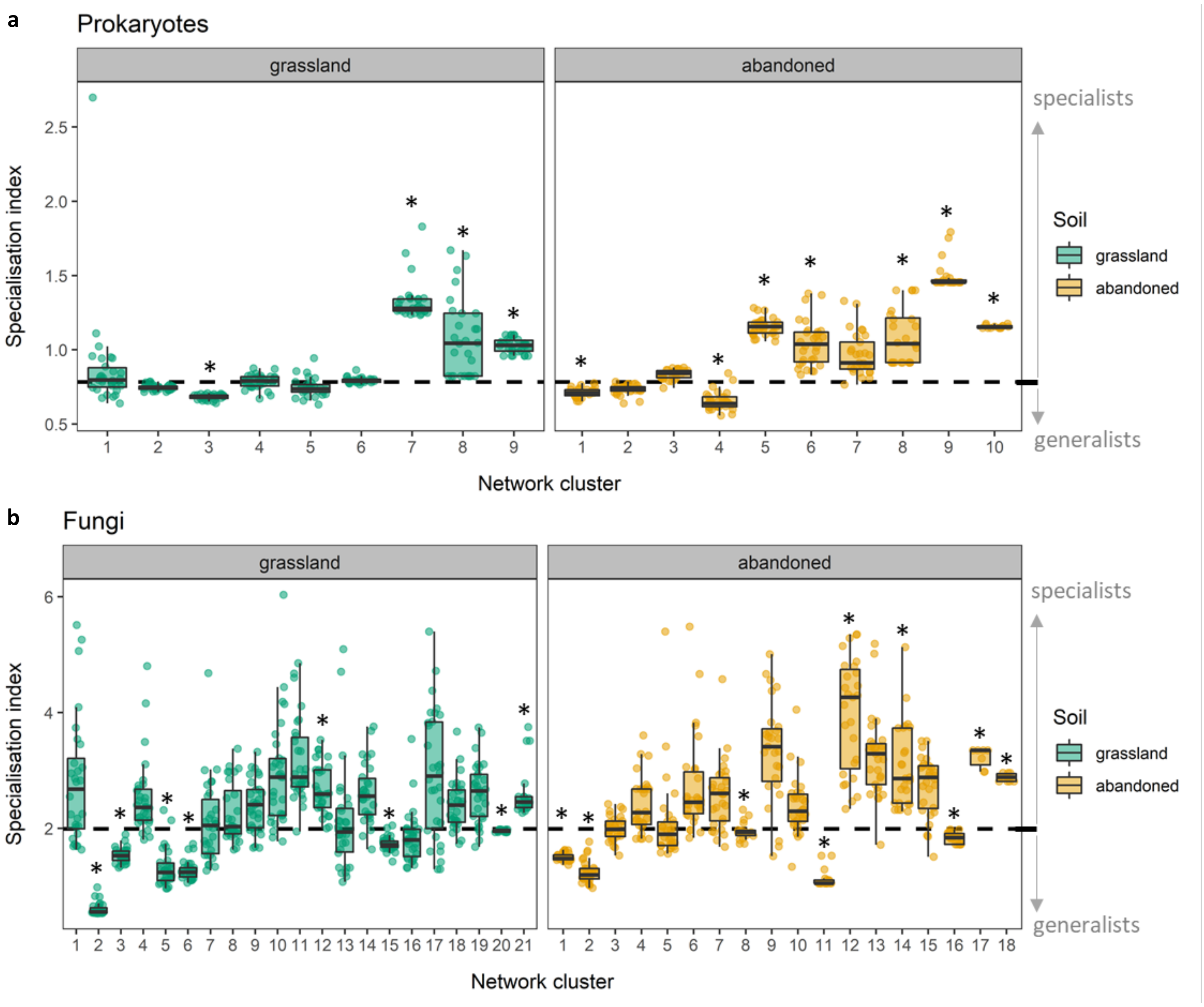

Supplementary Figure 9. **Relative habitat specialisation of network clusters.** Specialisation index (SI) of (**a**) prokaryote networks clusters and (**b**) fungal network clusters in natural grassland (green) and abandoned arable soil (yellow). The black, dashed line indicates the community-wide mean SI of the prokaryote and fungal community, respectively. Relative habitat specialist network clusters occur above the community-wide mean SI without overlap of the minimum distribution (25th percentile - 1.5 * interquartile rang; lower whisker). Relative habitat generalists network clusters occur below the community-wide mean SI without overlap of the maximum distribution (75th percentile + 1.5 * interquartile range; upper whisker). Relative habitat specialist and generalist clusters are indicated by an asterisk (*n* = 30 samples per cluster). Boxplots indicate median (middle line), 25th, 75th percentile (box) and 5th and 95th percentile (whiskers) with single points indicating each replicate. Source data are provided as a Source Data file.

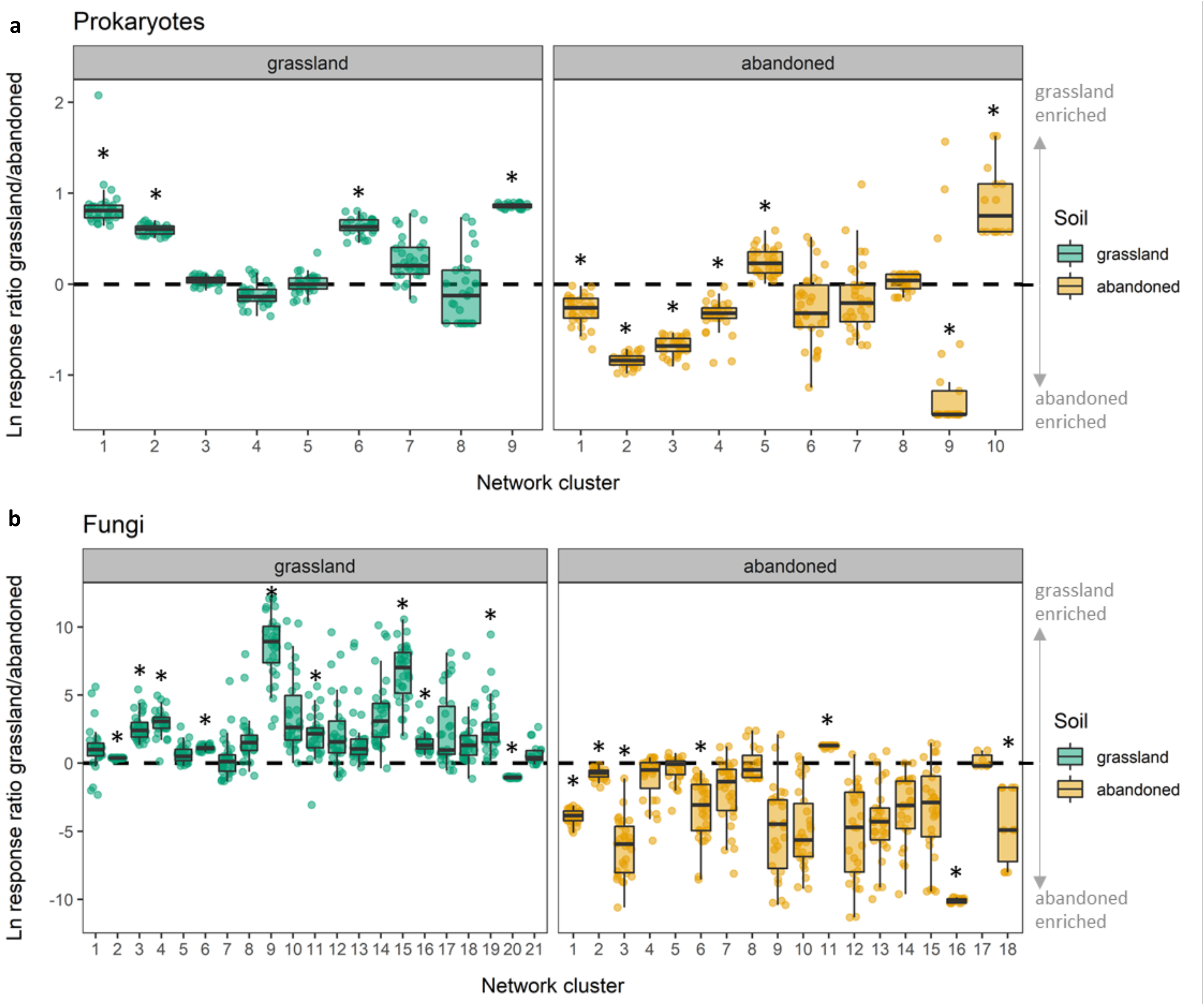

Supplementary Figure 10 **Network cluster enrichment in soils.** Ln-response ratio OTUs abundances in natural grassland soil divided by abundances in abandoned arable soil for (**a**) prokaryote networks clusters and (**b**) fungal network clusters in natural grassland (green) and abandoned arable soil (yellow). Clusters enriched in natural grassland soil occur above 0 (dashed line) without overlap of the minimum distribution (25th percentile - 1.5 * interquartile rang; lower whisker). Clusters enriched in abandoned arable soil occur below 0 without overlap of the maximum distribution (75th percentile + 1.5 * interquartile range; upper whisker). Enriched clusters are indicated by an asterisk (n = 30 samples per cluster). Boxplots indicate median (middle line), 25th, 75th percentile (box) and 5th and 95th percentile (whiskers) with single points indicating each replicate. Source data are provided as a Source Data file.

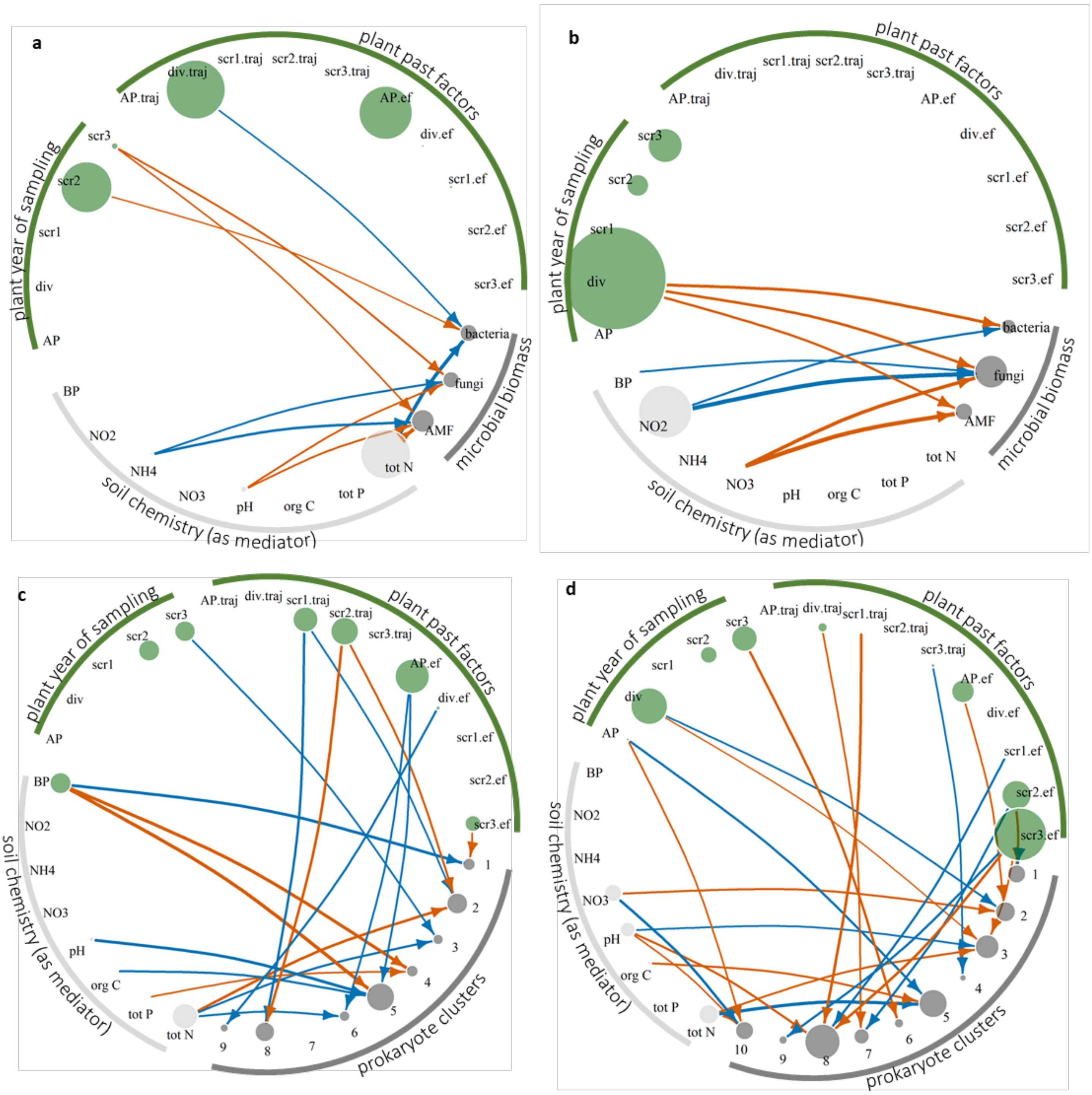

Supplementary Figure 11. Continued on next page.

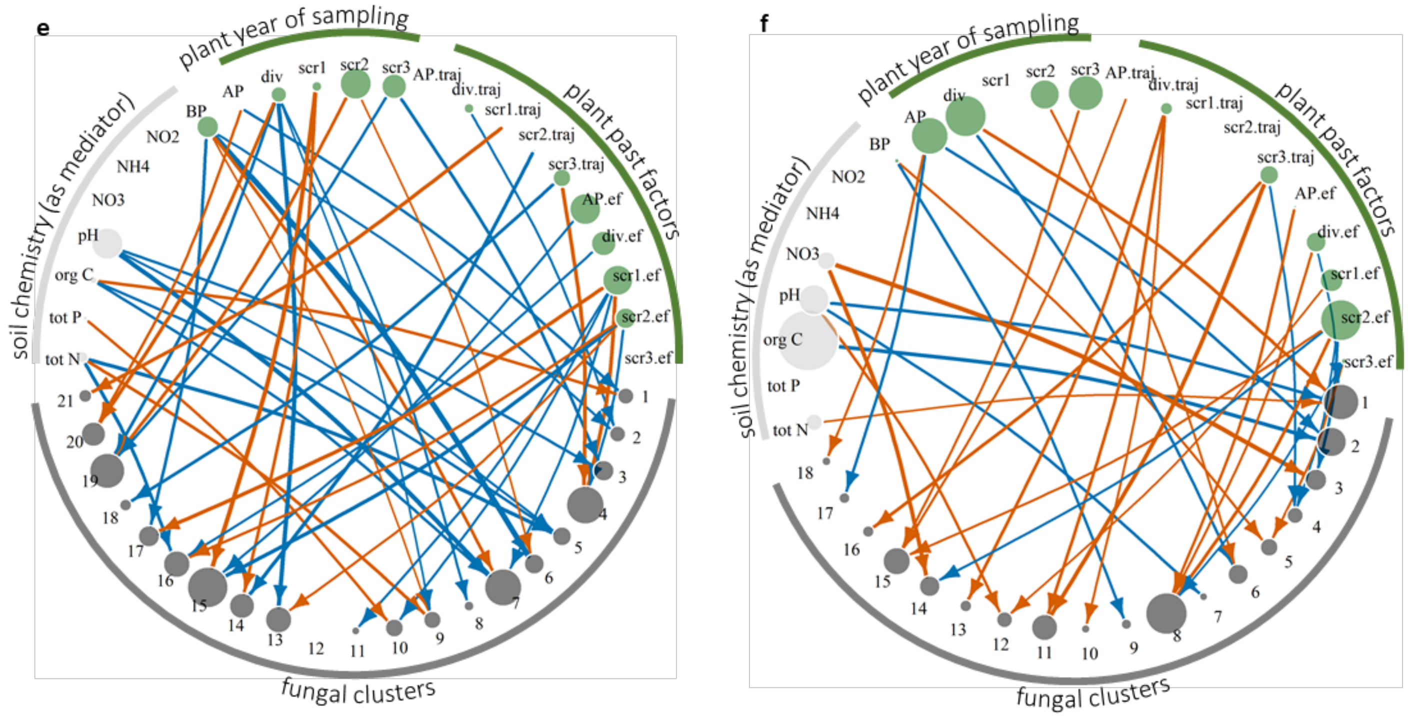

Supplementary Figure 11. **Plant-soil-microbial network cluster associations.** Significant pathways between plants (green), soil chemistry (light grey) and microbial parameters (dark grey) obtained from structural equation models in (**a, c, e**) natural grassland soil and (**b, d, f**) abandoned arable soil. Incorporated microbial parameters were (**a, b**) microbial biomass, (**b, d**) prokaryote network clusters and (**e, f**) fungal network clusters. Negative pathways are represented in vermillion, positive in blue. Arrows indicate the direction of the pathways and the width of the arrows its effect size. Plant vertice sizes indicate the summed direct and indirect pathway effect sizes onto microbial parameters. Soil chemical vertices indicate only the summed *in*direct pathway effect sizes. Microbial vertices indicate the summed direct and *in*direct pathway effect sizes that these microbial parameters were affected by. All summed pathway effect sizes were scaled to the size of the microbial parameters involved. For plant-soil chemical pathways, see Supplementary Fig. 12.

Plant year of sampling factors: AP – aboveground productivity, div – plant diversity, scr1 – plant composition DCA1 related to species residence period, scr2 – plant composition DCA2 related to species soil resource optima, scr3 – plant composition DCA3 related to community legume cover. Plant past factors: AP.traj – aboveground productivity trajectory, div.traj – plant diversity trajectory, scr1.traj – plant compositional DCA1 trajectory, scr2.traj – plant compositional DCA2 trajectory, scr3.traj – plant compositional DCA3 trajectory, AP.ef – effect size of the start of invasion on aboveground productivity, div.ef - effect size of the start of invasion on plant diversity, scr1.ef - effect size of the start of invasion on DCA1, scr2.ef - effect size of the start of invasion on DCA2, scr3.ef - effect size of the start of invasion on DCA3; soil chemistry: BP – belowground productivity. Only significant pathways included ($p < 0.05$; $n = 30$ communities). Source data are provided as a Source Data file.

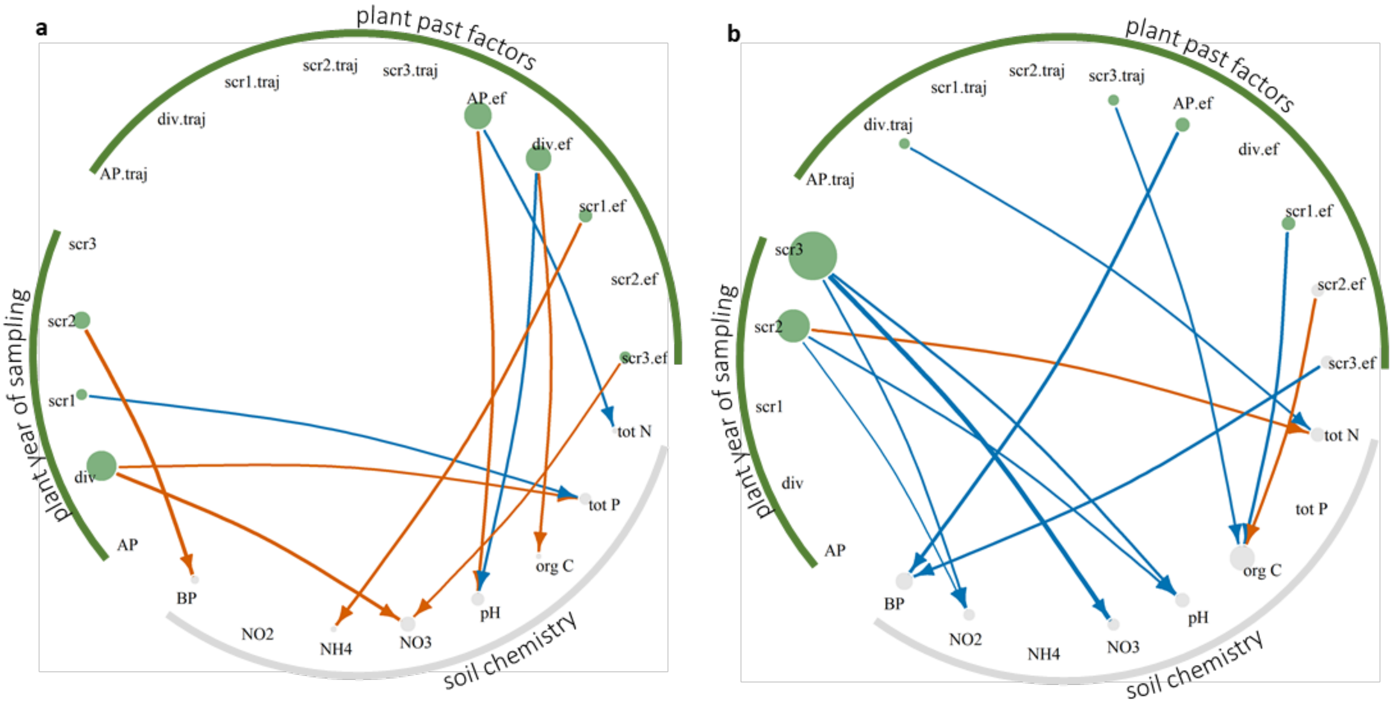

Supplementary Figure 12. **Plant-soil chemistry associations.** Significant pathways between plants (green) and soil chemistry (light grey) obtained from structural equation models in (**a**) natural grassland soil and (**b**) abandoned arable soil. Negative pathways are represented in vermillion, positive in blue. Arrows indicate the direction of the pathways and the width of the arrows its effect size. Plant vertice sizes indicate the summed pathway effect sizes of the parameter onto all soil chemical properties. Soil chemical vertice sizes indicate the summed pathway effect sizes of all plant community parameters the chemical parameter was affected by.

Plant year of sampling factors: AP – aboveground productivity, div – plant diversity, scr1 – plant composition DCA1 related to species residence period, scr2 – plant composition DCA2 related to species soil resource optima, scr3 – plant composition DCA3 related to community legume cover. Plant past factors: AP.traj – aboveground productivity trajectory, div.traj – plant diversity trajectory, scr1.traj – plant compositional DCA1 trajectory, scr2.traj – plant compositional DCA2 trajectory, scr3.traj – plant compositional DCA3 trajectory, AP.ef – effect size of the start of invasion on aboveground productivity, div.ef - effect size of the start of invasion on plant diversity, scr1.ef - effect size of the start of invasion on DCA1, scr2.ef - effect size of the start of invasion on DCA2, scr3.ef - effect size of the start of invasion on DCA3; soil chemistry: BP – belowground productivity. Only significant pathways included ($p < 0.05$; $n = 30$ communities). Source data are provided as a Source Data file.

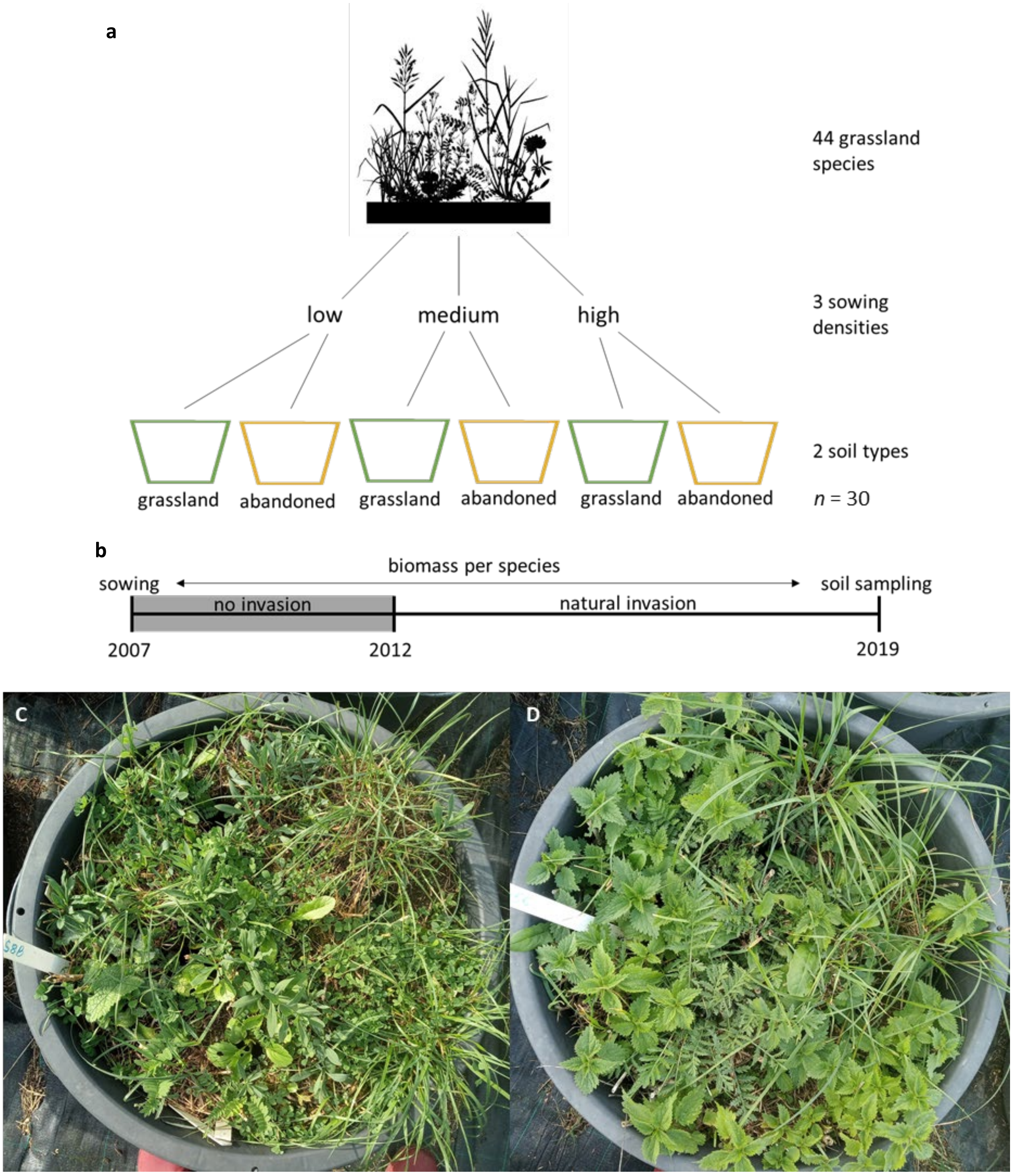

Supplementary Figure 13. **Experimental design. a** Experimental treatments. **b** Timeline of the experiment. Two typical images of a (**c**) natural grassland and (**d**) abandoned arable plant communities (summer 2020). In a, sowing densities represent 25% (low), 100% (medium) and 400% (high) of the natural seed densities as determined in a natural grassland community were the 44 plant species naturally coexist. Grassland soil was taken from a natural grassland and abandoned soil from an arable field that was abandoned in the 1950s (see also Münzbergová, 2012). In b, plant communities were sown in 2007 followed by a 5 year period in which invasion of other plant species was avoided. From 2012 to 2019, natural invasion occurred. In 2019 after the growing season, soil cores for chemical and microbial analysis were taken. For sown and invaded plant species, see Supplementary Table 9.

Supplementary Tables

Supplementary Table 1. **Microbial co-occurrence network parameters typically associated with network stability.**

	Prokaryote network		Fungal network	
	Natural grassland	Abandoned arable	Natural grassland	Abandoned arable
Number of nodes	1008	1024	403	455
Number of edges	10209	10079	1470	1874
Average number of edges per node	20.3	19.7	7.3	8.2
Negative edges	46%	46%	36%	33%
Edge betweenness	132	139	467	510
Average edge weight	0.07	0.07	0.08	0.08
Number of clusters	9	10	21	18
Clustering coefficient	0.36	0.35	0.54	0.56

Supplementary Table 2. **Dominant phyla, orders and families in soil prokaryote clusters of plant communities grown on natural grassland soil.**

Cluster	Dominant phyla (>20%)	Dominant orders (>9%)	Dominant families (>9%)	Relates to	Putative metabolic traits and functions
1 (95 OTUs)	*Proteobacteria (77.6%)*	*Sphingomondales (30.4%)*<br>*Pseudomonadales (18.6%)*<br>*Xanthomonadales (13.1%)*	*Sphingomonadaceae (30.3%)*<br>*Pseudomonadaceae (18.6%)*<br>*Xanthomonadaceae (13.1%)*	BP (+), invasion effect size DCA3 (-)	Abundant, ubiquitous soil and plant rhizosphere chemoheterotrophs; metabolically diverse, fast growing, various plant-phytopathogens and putative growth promoters[1–3]. Enriched in natural grassland soil.
2 (263 OTUs)	*Actinobacteria (57.7%)*<br>*Proteobacteria (32.6%)*	*Micrococcales (16.2%)*<br>*Solibrubacterales (12.2%)*<br>*Rhizobiales (11.1%)*	*Micrococcaceae (10.0%)*<br>*Solirubrobacteraceae (9.6%)*	Total N (-), DCA1 trajectory (+), DCA2 trajectory (-)	Diverse group of chemoheterotrophs, various involved in N-fixation[4,5]. Many unknown, enriched in natural grassland soil.
3 (244 OTUs)	*Bacteroidetes (44.6%)*<br>*Proteobacteria (26.9%)*	*Chitinophagales (24.9%)*<br>*Cytophagales (17.3%)*	*Chitinophagaceae (24.9%)*<br>*Fulvivirgaceae (16.3%)*	Total N (+), DCA3 (+)	Diverse group of generalist, chemoorganotrophs, degrading organic material, amongst others chitin[6,7]. Present in both soils.
4 (38 OTUs)	*Proteobacteria (80.6%)*	*Burkholderiales (38.0%)*<br>*Nitrosomonadales (13.3%)*	*Burkholderiaceae (33.9%)*<br>*Nitrosomonadaceae (13.3%)*	PO4 (-), BP (-)	Metabolically extremely diverse group including amongst others lithoautotrophic ammonia oxidizers[8,9]. Present in both soils.
5 (61 OTUs)	*Proteobacteria (66.1%)*	*Rhizobiales (27.6%)*<br>*Nevskiales (21.3%)*	*Steroidobacteraceae (21.3%)*<br>*Hyphomicrobiaceae (14.7%)*	Organic C (+), pH (+), BP (-), invasion effect size AP (+)	N-fixing taxa and taxa with unclear function[4]. Present in both soils.
6 (5 OTUs)	*Thaumarchaeota (98.7%)*	*Nitrososphaerales (98.7%)*	*Nitrososphaeraceae (98.7%)*	Total N (+), invasion effect size AP (+)	Ammonia oxidising archaea[10]. Enriched in natural grassland soil.
7 (120 OTUs)	*Proteobacteria (57.3%)*	*All below 9%*	*All below 9%*	Nothing	Unclear specialists. Present in both soils.
8 (2 OTUs)	*Actinobacteria (100%)*	*Acidimicrobiales (60.5%)*<br>*Streptomycetales (39.5%)*	*Acidimicrobiaceae (60.5%)*<br>*Streptomycetaceae (39.5%)*	DCA1 trajectory (+), DCA2 trajectory (-)	Specialists. Obligate acidophilic, reducing ferric iron (*Aciditerrimonas*) and common root endophytes producing mycelium and antibiotic secondary metabolites (*Streptomyces*)[11,12]. Present in both soils.
9 (2 OTUs)	*Actinobacteria (52.3%)*<br>*Proteobacteria (47.7%)*	*Thermoleophilales (52.3%)*<br>*Rhodobacterales (47.7%)*	*Thermoleophilaceae (52.3%)*<br>*Rhodobacteraceae (47.7%)*	Invasion effect size diversity (+)	Globally distributed specialists with unclear function[13]. Present in both soils.

Percentages indicate the relative read abundance of the phylogenetic group within the cluster. For cluster specialisation and enrichment metrices, see Supplementary Figs. 8-9.

Supplementary Table 3. **Dominant phyla, orders and families in soil prokaryote clusters of plant communities grown on abandoned arable soil.**

Cluster	Dominant phyla (>20%)	Dominant orders (>10%)	Dominant families (>9%)	Relates to	Putative metabolic traits and function
1 (232 OTUs)	*Proteobacteria (50.4%)*<br>*Actinobacteria (20.1%)*	*Spingomonadales (18.2%)*	*Sphingomonadaceae (16.8%)*	Invasion effect size DCA1 (-), invasion effect size DCA2 (+)	Diverse group of generalist, ubiquitous soil and plant rhizosphere chemoheterotrophs; metabolically diverse and putative growth promoters[3]. Many unknown, enriched in abandoned soil.
2 (226 OTUs)	*Proteobacteria (54.2%)*	*Burkholderiales (14.0%)*<br>*Chitinophagales (13.1%)*	*Chitinophagaceae (13.1%)*	NO3 (-), plant diversity (+), invasion effect size AP (-)	Diverse group of bacteria including chitin-degraders[6]. Many unknown, enriched in abandoned soil.
3 (245 OTUs)	*Proteobacteria (28.5%)*<br>*Actinobacteria (21.4%)*	*Nitrososphaerales (13.6%)*<br>*Rhizobiales (12.8%)*<br>*Cytophagales (10.6%)*	*Nitrososphaeraceae (13.6%)*<br>*Fulvivirgaceae (10.4%)*<br>*Hyphomicrobiaceae (9.9%)*	Total N (-), pH (+), plant diversity (-), invasion effect size DCA3 (-)	Diverse group of ammonia oxidising archaea, organic matter degraders and N-fixing taxa[4,10]. Enriched in abandoned soil.
4 (25 OTUs)	*Actinobacteria (44.2%)*<br>*Acidobacteria (25.7%)*<br>*Proteobacteria (22.2%)*	*Solirubrobacterales (15.8%)*<br>*Xanthomonadales (13.2%)*<br>*Gaiellales (12.5%)*	*Solirubrobacteraceae (15.8%)*<br>*Xanthomonadaceae (13.2%)*<br>*Gaiellaceae (12.5%)*	DCA3 trajectory (+)	Generalist, chemoheterotrophs, various plant-phytopathogens[2,14]. Enriched in abandoned soil.
5 (116 OTUs)	*Proteobacteria (52.0%)*<br>*Actinobacteria (22.2%)*	*Solirubrobacterales (12.4%)*<br>*Sphingomonadales (12.4%)*<br>*Rhizobiales (11.1%)*	*Solirubrobacteraceae (12.4%)*<br>*Sphingomonadaceae (12.0%)*<br>*Chitinophagaceae (9.0%)*	Total N (+), organic C (-), AP (+)	Specialist, ubiquitous soil and plant rhizosphere chemoheterotrophs including chitin-degraders; metabolically diverse and putative growth promoters[3]. Enriched in natural grassland soil.
6 (11 OTUs)	*Proteobacteria (60.5%)*	*Gemmatimonadales (17.3%)*<br>*Myxococcales (16.6%)*<br>*Burkholderiales (14.0%)*<br>*Micropepsales (12.8%)*<br>*Desulfuromonadales (11.9%)*<br>*Chthoniobacterales (11.0%)*	*Gemmatimonadaceae (17.3%)*<br>*Comamonadaceae (14.0%)*<br>*Micropepsaceae (12.8%)*<br>*Chthoniobacteraceae (11.0%)*	DCA3 (-)	Specialist, ubiquitous soil bacteria; metabolically diverse, including organic compound degraders and nitrate reducing bacteria[15–19]. Present in both soils.
7 (4 OTUs)	*Proteobacteria (45.9%)*<br>*Acidobacteria (27.4%)*	*Rhizobiales (45.9%)*<br>*Verrucomicrobiales (18.4%)*	*Methylocystaceae (45.9%)*<br>*Verrucomicrobia subdivision 3 (18.4%)*	Plant diversity trajectory (-), DCA2 trajectory (+)	Ubiquitous soil bacteria, including type II methanotrophs (aerobic methane oxidisers)[20,21]. Present in both soils.
8 (2 OTUs)	*Bacteroidetes (63.6%)*<br>*Acidobacteria (36.4%)*	*Chitinophagales (63.6%)*	*Chitinophagaceae (63.6%)*	pH (-), DCA1 trajectory (-), invasion effect size DCA1 (+), invasion effect size DCA3 (-)	Specialist, chitin-degraders[6]. Present in both soils.
9 (2 OTUs)	*Acidobacteria (76.8%)*	*Burkholderiales (14.8%)*	*Alcaligenaceae (14.8%)*	Invasion effect size DCA3 (+)	Unclear and many unknown specialists. Enriched in abandoned soil.
10 (1 OTU)	*Actinobacteria (68.0%)*<br>*Bacteroidetes (32.0%)*	*Acidimicrobiales (68.0%)*<br>*Chitinophagales (32.0%)*	*Acidimicrobiaceae (68.0%)*<br>*Chitinophagaceae (32.0%)*	pH (-), NO3 (+), AP (-)	Specialist, obligate acidophilic, ferric iron reducing bacteria (*Aciditerrimonas*) and chitin-degraders[6,12]. Enriched in natural grassland soil.

Percentages indicate the relative read abundance of the phylogenetic group within the cluster. For cluster specialisation and enrichment metrices, see Supplementary Figs. 8-9.

1. Roquigny, R., Novinscak, A., Biessy, A. & Filion, M. Pseudomonadaceae: From Biocontrol to Plant Growth Promotion. in *Rhizotrophs: Plant Growth Promotion to Bioremediation* 39–68 (Springer Singapore, 2017).
2. Ryan, R. P. *et al.* Pathogenomics of Xanthomonas: Understanding bacterium-plant interactions. *Nature Reviews Microbiology* **9** 344–355 (2011).
3. Glaeser, S. P. & Kämpfer, P. The Family Sphingomonadaceae. in *The Prokaryotes* 641–707 (Springer Berlin Heidelberg, 2014).
4. Jones, R. T. A Comprehensive Survey of Soil Rhizobiales Diversity Using High-Throughput DNA Sequencing. in *Biological Nitrogen Fixation* 769–776 (John Wiley & Sons, Inc, 2015).
5. Whitman, W. B. *Solirubrobacter*. in *Bergey's Manual of Systematics of Archaea and Bacteria* 1–5 (Wiley, 2015).
6. Wieczorek, A. S. *et al.* Ecological functions of agricultural soil bacteria and microeukaryotes in chitin degradation: A case study. *Front Microbiol* **10**, (2019).
7. Reichenbach, H. The Order Cytophagales. in *The Prokaryotes* 549–590 (Springer New York, 2006).
8. Coenye, T. The Family Burkholderiaceae. in *The Prokaryotes* 759–776 (Springer Berlin Heidelberg, 2014).
9. Prosser, J. I., Head, I. M. & Stein, L. Y. The Family Nitrosomonadaceae. in *The Prokaryotes* 901–918 (Springer Berlin Heidelberg, 2014).
10. Kerou, M. & Schleper, C. *Nitrososphaera*. in *Bergey's Manual of Systematics of Archaea and Bacteria* 1–10 (Wiley, 2016).
11. Kämpfer, P., Glaeser, S. P., Parkes, L., van Keulen, G. & Dyson, P. The Family Streptomycetaceae. in *The Prokaryotes* 889–1010 (Springer Berlin Heidelberg, 2014).
12. Stackebrandt, E. The Family Acidimicrobiaceae. in *The Prokaryotes* 5–12 (Springer Berlin Heidelberg, 2014).
13. Pujalte, M. J., Lucena, T., Ruvira, M. A., Arahal, D. R. & Macián, M. C. The Family Rhodobacteraceae. in *The Prokaryotes* 439–512 (Springer Berlin Heidelberg, 2014).
14. Albuquerque, L. & da Costa, M. S. The Family Gaiellaceae. in *The Prokaryotes* 357–360 (Springer Berlin Heidelberg, 2014).
15. Tang, S. *et al.* Microbial coupling mechanisms of nitrogen removal in constructed wetlands: A review. *Bioresour Technol* **314**, 123759 (2020).
16. Bhat, M. A. *et al.* Myxobacteria as a Source of New Bioactive Compounds: A Perspective Study. *Pharmaceutics* **13**, 1265 (2021).
17. Bräuer, S., Harbison, A. & Ueki, A. *Micropepsaceae*. in *Bergey's Manual of Systematics of Archaea and Bacteria* 1–5 (Wiley, 2018).

18. Hanada, S. & Sekiguchi, Y. The Phylum Gemmatimonadetes. in *The Prokaryotes* 677–681 (Springer Berlin Heidelberg, 2014).

19. Willems, A. The Family Comamonadaceae. in *The Prokaryotes* 777–851 (Springer Berlin Heidelberg, 2014).

20. White, R. A. *et al.* Moleculo Long-Read Sequencing Facilitates Assembly and Genomic Binning from Complex Soil Metagenomes. *mSystems* **1**, (2016).

21. Webb, H. K., Ng, H. J. & Ivanova, E. P. The Family Methylocystaceae. in *The Prokaryotes* 341–347 (Springer Berlin Heidelberg, 2014).

Supplementary Table 4. **Dominant phyla, orders, families and putative traits in soil fungal clusters of plant communities grown on natural grassland soil.**

Cluster	Dominant phyla (>20%)	Dominant orders (>9%)	Dominant families (>9%)	Relates to	Dominant fungal traits (>9%)	Putative metabolic traits and functions
1 (6 OTUs)	*Ascomycota (41.0%)*<br>*Mucoromycota (37.5%)*	*Mucorales (37.5%)*<br>*Archaeorhizomycetales (15.7%)*<br>*Mortierellales (15.0%)*	*Mucoraceae (37.5%)*<br>*Archaeorhizomycetaceae (15.7%)*<br>*Mortierellaceae (15.0%)*	Organic C (-), AP (+)	Soil saprotroph (68.2%)	Soil saprotrophs. Present in both soils.
2 (4 OTUs)	*Ascomycota (89.0%)*	*Filobasidiales (9.4%)*	*Piskurozymaceae (9.4%)*	BP (+), plant diversity trajectory (+)	Soil saprotroph (9.4%)	Abundant, generalist and largely unknown *Ascomycota*. Likely plant root associated. Enriched in natural grassland soil.
3 (46 OTUs)	*Mortierellomycota (50.7%)*<br>*Ascomycota (36.3%)*	*Mortierellales (50.7%)*	*Mortierellaceae (50.7%)*	pH (+), DCA3 (+)	Soil saprotroph (52.7%)<br>Wood saprotroph (10.9%)	Diverse group of generalist saprotrophs. Enriched in natural grassland soil.
4 (7 OTUs)	*Ascomycota (77.3%)*<br>*Basidiomycota (22.7%)*	*Archaeorhizomycetales (54.7%)*<br>*Agaricales (21.9%)*<br>*Geoglossales (18.1%)*	*Archaeorhizomycetaceae (54.6%)*<br>*Hygrophoraceae (19.2%)*<br>*Geoglossaceae (18.1%)*	DCA3 trajectory (-), invasion effect size DCA1 (-), invasion effect size DCA2 (+)	Soil saprotroph (94.7%)	Abundant soil saprotrophs. Enriched in natural grassland soil.
5 (17 OTUs)	*Ascomycota (52.5%)*<br>*Mortierellomycota (42.5%)*	*Mortierellales (42.5%)*<br>*Hypocreales (35.8%)*	*Mortierellaceae (42.5%)*<br>*Nectriaceae (33.4%)*	Total N (+), organic C (+), pH (+)	Plant pathogen (36.5%)<br>Soil saprotroph (35.4%)	Relative diverse and abundant, generalist soil saprotrophs and plant pathogens (*Fusarium, Ilyonectria, Verticillium, Leptosphaeria, Gibberella*). Present in both soils.
6 (13 OTUs)	*Ascomycota (82.2%)*	*Helotiales (57.5%)*<br>*Tremellales (16.5%)*<br>*Capnodiales (12.0%)*<br>*Thelebolales (11.2%)*	*Bulleribasidiaceae (16.5%)*<br>*Cladosporiaceae (12.0%)*<br>*Pseudeurotiaceae (11.2%)*	BP (+), DCA2 (-)	Soil saprotroph (27.9%)<br>Litter saprotroph (15.3%)	Relative abundant, generalist saprotrophs likely plant root associated. Enriched in natural grassland soil.
7 (10 OTUs)	*Ascomycota (86.4%)*	*Pezizales (50.1%)*<br>*Mortierellales (11.4%)*	*Pyronemataceae (46.3%)*<br>*Mortierellaceae (11.4%)*	Organic C (+), pH (+), BP (-), invasion effect size DCA1 (+)	Soil saprotroph (13.6%)<br>Plant pathogen (9.2%)	Soil saprotrophs and plant pathogens (*Fusarium*, *Lectera*). Present in both soils.
8 (5 OTUs)	*Ascomycota (69.5%)*<br>*Basidiomycota (23.7%)*	*Geoglossales (56.0%)*<br>*Agaricales (23.7%)*	*Geoglossaceae (56.0%)*<br>*Clavariaceae (23.7%)*	Plant diversity (+)	Soil saprotroph (79.7%)	Soil saprotrophs. Present in both soils.

Supplementary Table 4. Continued.

Cluster	Dominant phyla (>20%)	Dominant orders (>9%)	Dominant families (>9%)	Relates to	Dominant fungal traits	Putative function
9 (13 OTUs)	*Ascomycota (84.8%)*	*Archaeorhizomycetales (42.3%)*	*Archaeorhizomycetaceae (42.3%)*	Total N (-), BP (-), plant diversity (+)	Soil saprotroph (51.2%)	Soil saprotrophs. Enriched in natural grassland soil.
10 (19 OTUs)	*Ascomycota (55.8%)*	*Pleosporales (38.0%)* *Agaricales (13.5%)*	*Periconiaceae (21.0%)* *Hygrophoraceae (13.0%)*	PO4 (-), invasion effect size DCA1 (+)	Soil saprotroph (22.4%)	Diverse group of unknown fungi and soil saprotrophs. Present in both soils.
11 (11 OTUs)	*Ascomycota (64.1%)*	*Pleosporales (35.9%)* *Helotiales (14.3%)*	*Didymellaceae (21.2%)* *Sclerotiniaceae (13.8%)* *Pleosporaceae (11.2%)*	Invasion effect size DCA1 (+)	Plant pathogen (46.5%)	Plant pathogens (*Phoma, Botrytis, Stemphylium, Ophiosphaerella*). Enriched in natural grassland soil.
12 (5 OTUs)	*Mucoromycota (49.0%)* *Ascomycota (30.2%)*	*Mucorales (49.0%)* *Pleosporales (21.1%)*	*Mucoraceae (49.0%)* *Melanommataceae (21.1%)*	-	Soil saprotroph (50.4%) Plant pathogen (21.1%)	Specialist soil saprotrophs and plant pathogens (*Herpotrichia*). Present in both soils.
13 (8 OTUs)	*Ascomycota (81.0%)*	*Pleosporales (61.2%)*	*Phaeosphaeriaceae (32.2%)* *Pleosporales (24.5%)*	Plant diversity (+), invasion effect size DCA2 (-)	Plant pathogen (37.7%)	Plant pathogens (*Paraphoma, Septoria, Plenodomus*). Present in both soils.
14 (13 OTUs)	*Ascomycota (82.3%)*	*Pleosporales (33.0%)* *Geoglossales (19.7%)*	*Didymellaceae (25.6%)* *Geoglossaceae (19.7%)*	DCA1 (-), DCA2 trajectory (+)	Soil saprotroph (28.6%) Plant pathogen (26.7%)	Soil saprotrophs and plant pathogens (*Stagonosporopsis, Plectosphaerella*). Present in both soils.
15 (8 OTUs)	*Ascomycota (79.9%)*	*Chaetothyriales (41.7%)* *Pleosporales (30.7%)*	*Trichomeriaceae (37.3%)* *Didymellaceae (25.8%)*	DCA1 (-), invasion effect size AP (+), invasion effect size DCA2 (+)	Unspecified saprotroph (37.3%) Plant pathogen (36.3%)	Generalist saprotrophs and plant pathogens (*Ascochyta, Gibberella, Coniosporium*). Enriched in natural grassland soil.
16 (9 OTUs)	*Ascomycota (69.4%)*	*Pleosporales (44.0%)*	*Unidentified (76.3%)*	Total N (+), invasion effect size diversity (+), invasion effect size DCA2 (-)	Plant pathogen (11.1%)	Possible plant pathogens as order contains various putative plant pathogenic groups; also many unknown. Enriched in natural grassland soil.

Supplementary Table 4. Continued.

Cluster	Dominant phyla (>20%)	Dominant orders (>9%)	Dominant families (>9%)	Relates to	Dominant fungal traits	Putative function
17 (9 OTUs)	*Ascomycota (72.1%)* *Basidiomycota (25.9%)*	*Archaeorhizomycetales (58.2%)* *Trechisporales (12.2%)*	*Archaeorhizomycetaceae (58.2%)*	BP (+), invasion effect size DCA1 (-)	Soil saprotroph (62.7%) Wood saprotroph (9.1%)	Saprotrophs likely associated with plant roots. Present in both soils.
18 (10 OTUs)	*Ascomycota (32.3%)* *Basidiomycota (27.9%)*	*Sebacinales (18.5%)* *Mortierallales (14.1%)* *Rhizophlyctidales (9.9%)*	*Sebacinaceae (18.5%)* *Mortierellaceae (14.1%)* *Rhizophlyctidaceae (9.9%)*	DCA3 trajectory (+)	Litter saprotroph (22.8%) Soil saprotroph (14.1%)	Litter and soil saprotrophs. Present in both soils.
19 (13 OTUs)	*Ascomycota (57.4%)* *Basidiomycota (38.0%)*	*Hypocreales (21.0%)* *Agaricales (15.1%)* *Chaetothyriales (13.7%)* *Cantharellales (9.8%)*	*Ceratobasidiaceae (9.8%)*	Plant diversity (+), DCA2 (-), DCA3 (+)	Litter saprotroph (17.1%) Animal parasite (11.0%) Soil saprotroph (10.9%)	Relative diverse group of saprotrophs including putative nematode parasites. Enriched in natural grassland soil.
20 (1 OTU)	*Kickxellomycota (100%)*	*Kickxellales (100%)*	*Kickxellaceae (100%)*	AP (-), plant diversity (-)	Soil saprotroph (100%)	Generalist soil saprotrophs. Enriched in abandoned soil.
21 (2 OTUs)	*Ascomycota (54.9%)*	*Pleosporales (26.5%)* *Verrucariales (16.7%)* *Orbiliales (11.4%)*	*Phaeosphaeriaceae (26.5%)* *Verrucariaceae (16.7%)* *Orbiliaceae (11.4%)*	DCA1 trajectory (-)	Plant pathogen (26.5%) Lichenized (16.7%) Animal parasite (11.4%)	Specialist lichens, nematode parasites (*Arthrobotrys*) and plant pathogens (*Chaetosphaeronema*). Present in both soils.

Percentages indicate the relative read abundance of the phylogenetic group within the cluster. Dominant fungal traits were obtained from the FungalTrait database (Põlme, S. et al. FungalTraits: a user-friendly traits database of fungi and fungus-like stramenopiles. Fungal Diversity 105, (2020)). Genera identified as putative plant pathogens presented in brackets. For cluster specialisation and enrichment metrices, see Supplementary Figs. 8-9.

Supplementary Table 5. **Dominant phyla, orders, families and putative traits in soil fungal clusters of plant communities grown on abandoned arable soil.**

Cluster	Dominant phyla (>20%)	Dominant orders (>9%)	Dominant families (>9%)	Relates to	Dominant fungal traits	Putative function
1 (56 OTUs)	*Mortierellomycota (46.8%)*<br>*Ascomycota (43.8%)*	*Mortierellales (46.8%)*<br>*Pezizales (18.4%)*	*Mortierellaceae (46.8%)*<br>*Pyronemataceae (16.2%)*	Total N (-), pH (+), plant diversity (-), invasion effect size diversity (+)	Soil saprotroph (52.8%)	Diverse group of generalist soil saprotrophs. Enriched in abandoned soil.
2 (48 OTUs)	*Ascomycota (61.3%)*<br>*Mortierellomycota (34.8%)*	*Mortierellales (34.8%)*<br>*Hypocreales (18.7%)*	*Mortierellaceae (34.8%)*<br>*Nectriaceae (15.8%)*	Organic C (+), AP (+)	Soil saprotroph (38.5%)<br>Plant pathogen (17.8%)	Diverse group of generalist soil saprotrophs and plant pathogens (*Fusarium, Ilyonectria, Nectria, Plenodomus, Thielaviopsis, Lectera, Paraphoma, Ascochyta, Plectosphaerella, Stagonosporopsis*). Enriched in abandoned soil.
3 (27 OTUs)	*Ascomycota (46.3%)*<br>*Basidiomycota (42.3%)*	*Agaricales (35.7%)*<br>*Geoglossales (18.2%)*<br>*Archaeorhizomycetales (12.2%)*<br>*Incertae (9.8%)*	*Clavariaceae (33.0%)*<br>*Geoglossaceae (18.2%)*<br>*Archaeorhizomycetaceae (12.2%)*	NO3 (-), invasion effect size DCA2 (+)	Soil saprotroph (61.4%)	Diverse group of soil saprotrophs. Enriched in abandoned soil.
4 (5 OTUs)	*Ascomycota (62.4%)*<br>*Basidiomycota (37.6%)*	*Agaricales (37.6%)*<br>*Geoglossales (20.5%)*<br>*Eurotiales (14.2%)*<br>*Hypocreales (12.0%)*<br>*Saccharomycetales (11.6%)*	*Tricholomataceae (37.6%)*<br>*Geoglossaceae (20.5%)*<br>*Aspergillaceae (14.2%)*<br>*Nectriaceae (12.0%)*<br>*Debaryomycetaceae (11.6%)*	DCA3 trajectory (+), invasion effect size DCA2 (+)	Litter saprotroph (37.6%)<br>Soil saprotroph (20.5%)<br>Plant pathogen (16.1%)<br>Unspecified saprotroph (14.2%)<br>Nectar/tap saprotroph (11.6%)	Saprotrophs and plant pathogens (*Fusarium*, *Protomyces*). Present in both soils.
5 (17 OTUs)	*Ascomycota (48.9%)*<br>*Mortierellomycota (23.0%)*	*Hypocreales (34.1%)*<br>*Mortierellales (23.0%)*<br>*Glomerales (9.4%)*	*Mortierellaceae (23.0%)*<br>*Hypocreaceae (18.3%)*<br>*Nectriaceae (15.8%)*	BP (-), invasion effect size DCA3 (-)	Soil saprotroph (23.0%)<br>Mycoparasite (18.3%)	Diverse group of soil saprotrophs and mycoparasites. Present in both soils.

Supplementary Table 5. Continued.

Cluster	Dominant phyla (>20%)	Dominant orders (>9%)	Dominant families (>9%)	Relates to	Dominant fungal traits	Putative function
6 (39 OTUs)	*Ascomycota (56.3%)*<br>*Basidiomycota (21.3%)*	*Agaricales (17.4%)*<br>*Sordariales (15.0%)*<br>*Pleosporales (14.2%)*<br>*Orbiliales (12.4%)*	*Agaricaceae (17.1%)*<br>*Orbiliaceae (12.4%)*	Plant diversity (+), DCA2 (-)	Soil saprotroph (29.8%)	Diverse group of soil saprotrophs and small proportion, but high diversity of plant pathogens (*Herpotrichia, Chaetosphaeronema, Verticillium, Stemphylium, Periconia, Leptosphaeria, Fusarium, Alternaria, Gibellulopsis, Botrytis, Gibberella*). Enriched in abandoned soil.
7 (12 OTUs)	*Ascomycota (48.3%)*<br>*Basidiomycota (36.5%)*	*Sebacinales (21.9%)*<br>*Pezizales (19.5%)*<br>*Pleosporales (12.7%)*	*Sebacinaceae (21.9%)*<br>*Pyronemataceae (14.3%)*	pH (+)	Soil saprotroph (25.5%)<br>Litter saprotroph (11.6%)	Soil and litter saprotrophs. Present in both soils.
8 (2 OTUs)	*Kickxellomycota (57.4%)*	*Kickxellales (57.4%)*<br>*Mortierellales (11.9%)*	*Kickxellaceae (57.4%)*<br>*Mortierellaceae (11.9%)*	Invasion effect size AP (-), invasion effect size diversity (-), invasion effect size DCA2 (-), invasion effect size DCA3 (+)	Soil saprotroph (69.3%)	Generalist soil saprotrophs. Present in both soils.
9 (10 OTUs)	*Basidiomycota (42.1%)*<br>*Ascomycota (36.2%)*	*Sebacinales (29.1%)*	*Serendipitaceae (29.1%)*	BP (+)	Root endophyte (29.1%)<br>Litter saprotroph (10.9%)	Generalist root endophytes (*Serendipita*) and litter saprotrophs. Present in both soils.
10 (10 OTUs)	*Ascomycota (88.9%)*	*Pleosporales (39.8%)*<br>*Geoglossales (18.6%)*<br>*Pezizales (9.2%)*	*Didymellaceae (24.1%)*<br>*Geoglossaceae (18.6%)*<br>*Lentitheciaceae (9.2%)*<br>*Pyronemataceae (9.2%)*	Plant diversity trajectory (-)	Soil saprotroph (28.1%)<br>Plant pathogen (24.1%)<br>Root endophyte (9.2%)	Soil saprotrophs, plant pathogens (*Ascochyta*) and root endophytes (*Darksidea*). Present in both soils.
11 (2 OTUs)	*Chytridiomycota (100%)*	*Rhizophydiales (100%)*	*Rhizophydiaceae (100%)*	Plant diversity trajectory (-), DCA3 trajectory (-)	Algal parasite (100%)	Generalist algal parasites. Enriched in natural grassland soil.
12 (8 OTUs)	*Ascomycota (51.2%)*<br>*Olpidiomycota (36.3%)*	*Olpidiales (36.3%)*	*Olpidiaceae (36.3%)*	pH (-), DCA1 trajectory (-)	Algal parasite (36.3%)<br>Litter saprotroph (10.8%)	Specialist litter saprotrophs and algal parasites. Present in both soils.

Supplementary Table 5. Continued.

Cluster	Dominant phyla (>20%)	Dominant orders (>9%)	Dominant families (>9%)	Relates to	Dominant fungal traits	Putative function
13 (10 OTUs)	*Ascomycota (43.4%)*<br>*Basidiomycota (35.3%)*	*Agaricales (18.9%)*<br>*Mortierellales (15.3%)*<br>*Cantharellales (15.3%)*<br>*Pezizales (14.8%)*<br>*Hypocreales (10.4%)*	*Mortierellaceae (15.3%)*<br>*Cantharellaceae (15.3%)*<br>*Pyronemataceae (14.8%)*<br>*Marasmiaceae (12.8%)*	Plant diversity trajectory (-)	Soil saprotroph (17.2%)<br>Litter saprotroph (17.1%)<br>Lichen parasite (15.3%)<br>Wood saprotroph (14.3%)	Soil, litter and wood saprotrophs as well as lichen parasites. Present in both soils.
14 (6 OTUs)	*Ascomycota (50.4%)*<br>*Basidiomycota (37.7%)*	*Archaeorhizomycetales (44.6%)*<br>*Agaricales (37.7%)*<br>*Rhizophydiales (10.9%)*	*Archaeorhizomycetaceae (44.6%)*<br>*Clavariaceae (30.5%)*	NO3 (-), invasion effect size DCA2 (+)	Soil saprotroph (82.3%)	Specialist soil saprotrophs. Present in both soils.
15 (6 OTUs)	*Ascomycota (74.9%)*	*Chaetothyriales (19.9%)*<br>*Pleosporales (16.5%)*<br>*Helotiales (15.7%)*<br>*Orbiliales (10.8%)*<br>*Cystofilobasidiales (10.3%)*	*Sporormiaceae (16.5%)*<br>*Orbiliaceae (10.8%))*<br>*Mrakiaceae (10.3%)*	DCA3 (-), AP trajectory (-), invasion effect size DCA2 (-)	Dung saprotroph (16.5%)<br>Plant pathogen (10.3%)<br>Litter saprotroph (9.9%)	Litter and dung saprotrophs, and plant pathogens (*Itersonilia*). Present in both soils.
16 (2 OTUs)	*Basidiomycota (100%)*	*Unidentified (100%)*	*Unidentified (100%)*	DCA3 trajectory (-)	Unknown (100%)	Unknown generalists. Enriched in abandoned soil.
17 (1 OTU)	*Ascomycota (100%)*	*Melanosporales (60.6%)*	*Melanosporaceae (60.6%)*	AP (+)	Mycoparasite (60.6%)	Specialist mycoparasites. Present in both soils.
18 (1 OTU)	*Basidiomycota (53.3%)*<br>*Ascomycota (46.7%)*	*Sebacinales (53.3%)*<br>*Helotiales (46.7%)*	*Serendipitaceae (53.3%)*	AP (-)	Root endophyte (53.3%)	Specialist root endophytes (*Serendipita*). Enriched in abandoned soil.

Percentages indicate the relative read abundance of the phylogenetic group within the cluster. Dominant fungal traits were obtained from the FungalTrait database (Põlme, S. et al. FungalTraits: a user-friendly traits database of fungi and fungus-like stramenopiles. Fungal Diversity 105, (2020)). Genera identified as putative plant pathogens presented in brackets. For cluster specialisation and enrichment metrices, see Supplementary Figs. 8-9.

Supplementary Table 6. **Marginal R^2 of soil chemistry, microbial biomass and clusters from SEM models in natural grassland and abandoned arable plant communities.**

	Natural grassland	Abandoned arable		Natural grassland	Abandoned arable
Soil chemistry			Fungal clusters		
BP	0.31	0.43	1	0.34	**0.59**
Total N	0.18	0.24	2	**0.29**	**0.41**
Total P	0.21	0.00	3	**0.34**	**0.38**
Organic C	0.16	0.50	4	**0.44**	0.20
NO3	0.43	0.47	5	**0.41**	0.34
NH4	0.21	0.00	6	**0.61**	0.28
NO2	0.00	0.19	7	0.53	0.15
pH	0.26	0.25	8	0.15	0.38
PLFA/NLFA			9	0.61	0.18
Bacterial biomass	0.52	0.49	10	0.31	0.12
Fungal biomass	0.41	0.54	11	0.10	0.28
AMF biomass	0.41	0.48	12	0.00	0.20
Prokaryote clusters			13	0.35	0.22
1	**0.30**	**0.35**	14	0.33	0.36
2	**0.41**	**0.54**	15	0.62	0.39
3	**0.29**	**0.64**	16	0.36	0.20
4	0.36	0.12	17	0.33	0.18
5	0.35	0.4	18	0.21	0.13
6	0.4	0.22	19	0.29	
7	0.00	0.29	20	0.15	
8	0.23	0.53	21	0.21	
9	0.17	0.16			
10		0.35			

R^2 in bold belong to large clusters (Fig. 3). Note: sowing density was incorporated as a random effect, but in most cases explained no variation. Marginal R^2 indicates variation explained by fixed factors only.

Supplementary Table 7. **Summarised putative effects of the strongest pathways of the plant community in the year of sampling and past on microbial communities in natural grassland soil.**

Time point	Plant parameter	Pathway	Overall microbial parameters	Prokaryote clusters	Fungal clusters	Putative metabolic traits and functions
Year of sampling	AP	direct	-	-	↑1 ↓20	↑ Soil saprotrophs<br>↓ Generalist soil saprotrophs
**Year of sampling**	**Plant diversity**	**direct**	↑fungal diversity	-	↑8 ↑9 ↑13 ↑19 ↓20	↑ Metabolically diverse group of bacteria including ammonia oxidizers; diverse group of soil saprotrophs, various plant pathogens, nematode parasites and diverse group unknown fungi<br>↓ Generalist soil saprotrophs
		via ↓P	↑fungal diversity	↑4	↑10	
Year of sampling	Composition (DCA1) – temporal turnover	direct	↓prokaryote SI	-	↓14 ↓15	↓ Metabolically diverse group of bacteria including ammonia oxidizers; diverse group of unknown fungi, soil saprotrophs and plant pathogens; generalist saprotrophs and plant pathogens
		via ↑P	↓fungal diversity	↓4	↓10	
**Year of sampling**	**Composition (DCA2) – soil resource optima**	direct	↓bacterial biomass<br>↓fungal diversity	-	**↓6** ↓19	↑ Metabolically diverse group of bacteria including ammonia oxidizers and N-fixers; soil saprotrophs and plant pathogens<br>↓ Bacterial biomass; ubiquitous soil and plant rhizosphere chemoheterotrophs; abundant, generalist saprotrophs and unknown fungi (likely root-associated); diverse group of soil saprotrophs
		**via ↓BP**	↑prokaryote diversity	**↓1** ↑4 ↑5	**↓2** **↓6** ↑7 ↑9 ↓17	
**Year of sampling**	**Composition (DCA3) – legume cover**	**direct**	↓fungal biomass<br>↓AMF biomass	**↑3**	**↑3** ↑19	↑ Generalist, chemoorganotrophs including chitin degrading bacteria; diverse group of generalist saprotrophs<br>↓ Fungal and AMF biomass
Past	AP trajectory	direct	-	-	-	-
Past	Plant diversity trajectory	direct	↑bacterial biomass	-	-	↑ Bacterial biomass.
**Past**	**2012 invasion effect size AP**	direct	↑fungal diversity	↑5 ↑6	↑15	↑ Bacterial, fungal and AMF biomass; ammonia oxidising archaea; N-fixing and diverse group of generalist organic compound degrading bacteria; diverse and abundant, generalist soil saprotrophs and plant pathogens.<br>↓ AMF biomass; N-fixing and diverse group of chemoheterotrophic bacteria; diverse and abundant, generalist soil saprotrophs and plant pathogens.
		**via ↑N**	↑bacterial biomass<br>↓AMF biomass<br>↑prokaryote SI	**↓2** **↑3** ↑6	**↑5** ↓9 ↑16	
		**via ↓pH**	↑fungal biomass<br>↑AMF biomass	↓5	**↓3** **↓5** ↓7	
**Past**	**2012 invasion effect size diversity**	direct	↓fungal SI	↑9	↑16	↑ N-fixing and ubiquitous specialist bacteria; diverse group of soil saprotrophs and plant pathogens<br>↓ Fungal and AMF biomass; N-fixing bacteria; some soil saprotrophs and plant pathogens
		**via ↓org C**	↑prokaryote SI	↓5	↑1 **↓5** ↓7	
		**via ↑pH**	↓fungal biomass<br>↓AMF biomass	↑5	**↑3** **↑5** ↑7	

Pathways in bold indicate pathways with a relative contribution > 5% (Fig. 6). Clusters in bold indicate dominant clusters (Fig. 3). Clusters with a thick underline were classified as relative habitat generalists and clusters with a wave underline were classified as relative habitat specialists (Supplementary Fig. 9). Clusters highlighted in green were enriched in natural grassland soil and clusters highlighted in red were enriched in abandoned arable soil (Supplementary Fig. 10). For cluster specific information, see Supplementary Tables 2 and 4.

Supplementary Table 7. Continued.

Time point	Plant parameter	Pathway	Overall microbial parameters	Prokaryote clusters	Fungal clusters	Putative metabolic traits and functions
Past	**DCA1 trajectory**	**direct**	-	**↑2** ↑8	↓21	↑ Diverse group of chemoheterotrophs, various involved in N-fixation; specialist obligate acidophiles and root endophytes<br>↓ Specialist lichens, nematode parasites and plant pathogens
Past	**DCA2 trajectory**	**direct**	-	**↓2** ↓8	↑14	↑ Soil saprotrophs and plant pathogens<br>↓ Diverse group of chemoheterotrophs, various involved in N-fixation; specialist obligate acidophiles and root endophytes
Past	**DCA3 trajectory**	**direct**	-	-	**↓4** ↑18	↑ Litter and soil saprotrophs.<br>↓ Abundant soil saprotrophs.
Past	**2012 invasion effect size DCA1**	**direct**	-	-	**↓4** ↑7 ↑10 ↑11 ↓17	↑ Diverse group of unknown fungi, soil saprotrophs and plant pathogens.<br>↓ Fungal and AMF biomass; abundant soil saprotrophs.
		via ↓NH4	↓fungal biomass ↓AMF biomass	-	-	
Past	**2012 invasion effect size DCA2**	**direct**	-	-	**↑4** ↓13 ↑15 ↓16	↑ Abundant soil saprotrophs, generalist saprotrophs and plant pathogens.<br>↓ Soil saprotrophs and plant pathogens.
Past	**2012 invasion effect size DCA3**	**direct**	-	**↓1**	-	↓ Abundant ubiquitous soil and plant rhizosphere chemoheterotrophs

Pathways in bold indicate pathways with a relative contribution > 5% (Fig. 6). Clusters in bold indicate dominant clusters (Fig. 3). Clusters with a thick underline were classified as relative habitat generalists and clusters with a wave underline were classified as relative habitat specialists (Supplementary Fig. 9). Clusters highlighted in green were enriched in natural grassland soil and clusters highlighted in red were enriched in abandoned arable soil (Supplementary Fig. 10). For cluster specific information, see Supplementary Tables 2 and 4.

Supplementary Table 8. **Summarised putative effects of the strongest pathways of the plant community in the year of sampling and past on microbial communities in abandoned arable soil.**

Time point	Plant parameter	Pathway	Overall microbial parameters	Prokaryote clusters	Fungal clusters	Putative metabolic traits and functions
Year of sampling	**Aboveground productivity**	**direct**	-	↑5 ↓10	**↑2** ↑17 ↓18	↑ Specialist, ubiquitous soil and plant rhizosphere chemoheterotrophs including chitin-degraders; diverse group of generalist soil saprotrophs and plant pathogens; specialist mycoparasites<br>↓ Specialist, obligate acidophiles and chitin-degraders; specialist root endophytes
Year of sampling	**Plant diversity**	**direct**	↓bacterial biomass<br>↓fungal biomass<br>↓AMF biomass	**↑2 ↓3**	**↓1** ↑6	↑ Broad group of bacteria including chitin-degraders; diverse group of saprotrophs and plant pathogens<br>↓ Bacterial, fungal and AMF biomass; diverse group of ammonia oxidising archaea, organic matter degraders and N-fixing taxa; diverse group of generalist soil saprothrophs
Year of sampling	Composition (DCA1) – temporal turnover	direct	-	-	-	-
Year of sampling	**Composition (DCA2) – soil resource optima**	direct	-	-	↓6	↑ Diverse group of ammonia oxidising archaea, organic matter degraders and N-fixing taxa; diverse group of generalist soil saprotrophs<br>↓ Specialist, ubiquitous soil and plant rhizosphere chemoheterotrophs; specialist chitin-degraders and obligate acidophiles; diverse group of soil saprotrophs and plant pathogens; specialist litter saprotrophs and algal parasites
		**via ↓N**	-	**↑3** ↓5	**↑1**	
		**via ↑pH**	↓fungi SI	**↑3** ↓8 ↓10	**↑1** ↑7 ↓12	
Year of sampling	**Composition (DCA3) – legume cover**	direct	↓AMF biomass	↓6	↓15	↑ Diverse group of ammonia oxidising archaea, organic matter degraders and N-fixing taxa; diverse group of generalist saprotrophs<br>↓ Fungal and AMF biomass; abundant, broad group of bacteria including chitin-degraders; specialist, ubiquitous soil bacteria including organic compound and chitin degraders, nitrate reducing and obligate acidophiles; diverse group of soil saprotrophs; specialist saprotrophs and algal parasites; plant pathogens
		**via ↑NO3**	↓fungal biomass<br>↓AMF biomass	**↓2**	**↓3** ↓14	
		**via ↑pH**	↓fungi SI	**↑3** ↓8 ↓10	**↑1** ↑7 ↓12	
Past	AP trajectory	direct	-	-	↓15	↓ litter and dung saprotrophs, and plant pathogens
Past	Plant diversity trajectory	direct	-	↓7	-	↑ Specialist, ubiquitous soil and plant rhizosphere chemoheterotrophs.<br>↓ Diverse group of ammonia oxidising archaea, organic matter degraders, N-fixing taxa and methanotrophs; diverse group of generalist soil saprotrophs.
		via ↑N	-	**↓3** ↑5	**↓1**	
Past	**2012 invasion effect size AP**	**direct**	↓prokaryote SI	**↓2**	↓8	↑ Fungal biomass; generalist root endophytes and litter saprotrophs.<br>↓ Diverse group of bacteria including chitin-degraders; diverse group of soil saprotrophs and mycoparasites; generalists soil saprotrophs.
		via ↑BP	↑fungal biomass	-	↓5 ↑9	
		via ↓NO2	↓bacterial biomass<br>↓fungal biomass	-	-	

Pathways in bold indicate pathways with a relative contribution > 5% (Fig. 6). Clusters in bold indicate dominant clusters (Fig. 3). Clusters with a thick underline were classified as relative habitat generalists and clusters with a wave underline were classified as relative habitat specialists (Supplementary Fig. 9). Clusters highlighted in green were enriched in natural grassland soil and clusters highlighted in red were enriched in abandoned arable soil (Supplementary Fig 10). For cluster specific information, see Supplementary Tables 3 and 5.

Supplementary Table 8. Continued.

Time point	Plant parameter	Pathway	Overall microbial parameters	Prokaryote clusters	Fungal clusters	Putative metabolic traits and functions
Past	2012 invasion effect size diversity	direct	-	-	↑4 ↓8	↑ Saprotrophs and plant pathogens.<br>↓ Bacterial and fungal biomass; generalist soil saprotrophs.
		via ↓NO2	↓bacterial biomass<br>↓fungal biomass	-	-	
Past	DCA1 trajectory	direct	-	↓8	↓10 ↓11<br>↓13	↓ Specialist chitin-degraders; soil, wood, litter saprotrophs, plant pathogens and root endophytes, generalist algal parasites.
Past	DCA2 trajectory	direct	-	-	-	-
Past	**DCA3 trajectory**	direct	-	↑4	↑4 ↓11<br>↓16	↑ Generalist chemoheterotrophs, various plant-phytopathogens; diverse group of generalist soil saprotrophs and plant pathogens.<br>↓ Specialist, ubiquitous soil and plant rhizosphere chemoheterotrophs; generalist algal parasites and unkown fungi.
		**via ↑orgC**	-	↓5	**↑2**	
Past	**2012 invasion effect size DCA1**	direct	-	↑8	↓12	↑ Specialist chitin-degraders; diverse group of generalist soil saprotrophs and plant pathogens.<br>↓ Specialist, ubiquitous soil and plant rhizosphere chemoheterotrophs; specialist litter saprotrophs and algal parasites.
		**via ↑orgC**	-	↓5	**↑2**	
Past	**2012 invasion effect size DCA2**	**direct**	↑prokaryote diversity	**↓1** ↑7	**↑1** ↓8<br>↑14 ↓15	↑ Specialist, ubiquitous soil and plant rhizosphere bacteria, including methanotrophs and chitin-degraders; diverse group of generalist and specialist soil saprotrophs.<br>↓ Diverse group of generalist, ubiquitous soil and plant rhizosphere chemoheterotrophs; diverse group of generalist soil saprotrophs and plant pathogens; litter and dung saprotrophs.
		**via ↓orgC**	-	↑5	**↓2**	
Past	**2012 invasion effect size DCA3**	**direct**	↓prokaryote diversity<br>↓fungal diversity	**↑1** **↓3** ↓8<br>↑9	↓5 ↑8	↑ Diverse group of generalist ubiquitous soil and plant rhizosphere chemoheterotrophs, unknown specialist bacteria; generalist soil saprotrophs.<br>↓ Diverse group of ammonia oxidising archaea, organic matter degraders and N-fixing taxa; specialist chitin-degraders; diverse group of soil saprotrophs and mycoparasites.

Pathways in bold indicate pathways with a relative contribution > 5% (Fig. 6). Clusters in bold indicate dominant clusters (Fig. 3). Clusters with a thick underline were classified as relative habitat generalists and clusters with a wave underline were classified as relative habitat specialists (Supplementary Fig. 9). Clusters highlighted in green were enriched in natural grassland soil and clusters highlighted in red were enriched in abandoned arable soil (Supplementary Fig 10). For cluster specific information, see Supplementary Tables 3 and 5.

Supplementary Table 9. **Sown and invaded plant species and their abbreviations.**

Plant species	Abbreviation	Sown/invaded
Acer spp	Acesp	Invaded
Agrimonia eupatorium	Agreu	Sown
Agrostis spp	Agrsp	Invaded
Anthericum ramosum	Antra	Sown, not established
Anthylis vulneraria	Antvu	Sown
Arabidopsis thaliana	Arath	Invaded
Arenaria serpyllifolia	Arese	Invaded
Arrhenatherum elatior	Arrel	Invaded
Artemisia vulgaris	Artvu	Invaded
Asperula spp	Aspsp	Sown
Aster amellus	Astam	Sown, not established
Astragalus cicer	Astci	Sown
Astragalus glycyphylos	Astgl	Sown
Atriplex spp	Atrsp	Invaded
Brachypodium pinnatum	Brapi	Sown
Bromus erectus	Broer	Sown
Bromus mollis	Bromo	Invaded
Bupleurum falcatum	Bupfa	Sown
Calamagrostis epigejos	Calep	Invaded
Campanula gentilis	Camge	Sown
Campanula glomerata	Camgl	Sown
Campanula patula	Campa	Invaded
Carex flacca	Carfl	Sown
Carex hirta	Carhi	Invaded
Cardamine spp	Carsp	Invaded
Carex tomentosa	Carto	Sown
Centaurea jacea	Cenja	Sown
Centaurea scabiosa	Censc	Sown
Cerastium holosteoides	Cerho	Invaded
Cirsium acaule	Cirac	Sown, not established
Cirsium pannonicum	Cirpa	Sown
Conyza spp	Consp	Invaded
Coronila varia	Corva	Sown, not established
Crepis biennis	Crebi	Invaded
Dactylis glomerata	Dacgl	Invaded
Daucus carota	Dauca	Invaded
Dianthus carthusianorum	Diaca	Sown, not established
Dipsacus sylvestris	Dipsy	Invaded
Elymus repens	Elyre	Invaded
Epilobium spp	Episp	Invaded
Erigeron annuus	Erian	Invaded
Euphorbia cyparissias	Eupcy	Invaded
Fallopia convolvulus	Falco	Invaded
Falcaria vulgaris	Falvu	Invaded
Galeopsis spp	Galsp	Invaded
Geranium sibiricum	Gersi	Invaded
Helianthemum grandiflorum	Helgr	Sown
Heracleum mantegazzianum	Herma	Invaded
Hieracium spp	Hiesp	Invaded
Holcus mollis	Holmo	Invaded
Hypericum perforatum	Hyppe	Invaded
Inula hirta	Inuhi	Sown
Inula salicina	Inusa	Sown

Supplementary Table 9. Continued.

Plant species	Abbreviation	Sown/invaded
Lactuca serriola	Lacse	Invaded
Lamium purpureum	Lampu	Invaded
Laserpitium latifolium	Lasla	Sown
Lathyrus pratensis	Latpr	Invaded
Leontodon autumnalis	Leoau	Invaded
Leontodon hispidus	Leohi	Sown
Linum catharticum	Linca	Invaded
Linum flavum	Linfl	Sown
Linum tenuifolium	Linte	Sown
Lolium perenne	Lolpe	Invaded
Lotus corniculatus	Lotco	Sown
Medicago falcata	Medfa	Sown
Medicago lupulina	Medlu	Invaded
Myos spp	Myosp	Invaded
Plantago lanceolata	Plala	Invaded
Plantago media	Plame	Sown
Poa annua	Poaan	Invaded
Poa trivialis	Poatr	Invaded
Polygonum spp	Polsp	Invaded
Primula veris	Prive	Sown, not established
Prunella grandiflora	Prugr	Sown
Ranunculus spp	Ransp	Invaded
Raphanus raphanistrum	Rapra	Invaded
Rumex spp	Rumsp	Invaded
Salvia pratensis	Salpr	Sown
Salix spp	Salsp	Invaded
Salvia verticilata	Salve	Sown
Sanquisorba minor	Sanmi	Sown
Scabiosa ochroleuca	Scaoc	Sown
Silene vulgaris	Silvu	Invaded
Solidago canadensis	Solca	Invaded
Sonchus spp	Sonsp	Invaded
Stachys recta	Stare	Sown
Stipa spp	Stisp	Invaded
Tanacetum corymbosum	Tanco	Sown
Tanacetum spp	Tansp	Invaded
Tanacetum vulgaris	Tanvu	Invaded
Taraxacum officinalis	Tarof	Invaded
Teucrium chamaedrys	Teuch	Sown
Thlaspi arvense	Thlar	Invaded
Thymus pulegioides	Thypu	Sown
Trifolium medium	Trime	Sown
Trifolium montanum	Trimo	Sown
Trifolium pratensis	Tripr	Invaded
Trifolium repens	Trire	Invaded
Tussilago farfara	Tusfa	Invaded
Urtica dioica	Urtdi	Invaded
Veronica teucrium	Verteu	Sown
Viccia spp	Vicsp	Invaded
