# Supplementary material for: Plant community stability is associated with a decoupling of prokaryote and fungal soil networks
Source: Nat Commun. 2023 Jun 22;14:3736. doi: 10.1038/s41467-023-39464-8 (PMC10287681; doi:10.1038/s41467-023-39464-8)
Supplement: Supplementary file 2 — Reporting Summary [file 41467_2023_39464_MOESM2_ESM.pdf]

nature portfolio

Corresponding author(s): Dina in 't Zandt

Last updated by author(s): 14/06/2023

Reporting Summary

Nature Portfolio wishes to improve the reproducibility of the work that we publish. This form provides structure for consistency and transparency in reporting. For further information on Nature Portfolio policies, see our Editorial Policies and the Editorial Policy Checklist.

Statistics

For all statistical analyses, confirm that the following items are present in the figure legend, table legend, main text, or Methods section.

n/a	Confirmed	
☐	☒	The exact sample size (*n*) for each experimental group/condition, given as a discrete number and unit of measurement
☐	☒	A statement on whether measurements were taken from distinct samples or whether the same sample was measured repeatedly
☐	☒	The statistical test(s) used AND whether they are one- or two-sided<br>*Only common tests should be described solely by name; describe more complex techniques in the Methods section.*
☐	☒	A description of all covariates tested
☐	☒	A description of any assumptions or corrections, such as tests of normality and adjustment for multiple comparisons
☐	☒	A full description of the statistical parameters including central tendency (e.g. means) or other basic estimates (e.g. regression coefficient) AND variation (e.g. standard deviation) or associated estimates of uncertainty (e.g. confidence intervals)
☐	☒	For null hypothesis testing, the test statistic (e.g. *F*, *t*, *r*) with confidence intervals, effect sizes, degrees of freedom and *P* value noted<br>*Give P values as exact values whenever suitable.*
☐	☒	For Bayesian analysis, information on the choice of priors and Markov chain Monte Carlo settings
☐	☒	For hierarchical and complex designs, identification of the appropriate level for tests and full reporting of outcomes
☒	☐	Estimates of effect sizes (e.g. Cohen's *d*, Pearson's *r*), indicating how they were calculated

Our web collection on statistics for biologists contains articles on many of the points above.

Software and code

Policy information about availability of computer code

Data collection	No software was used to collect data
Data analysis	16S and ITS amplicons were analysed in the SEED2 version. 2.1.1b pipeline (Větrovský et al. 2018, Bioinformatics 34, 2292–2294). All other data analyses were performed in R version 3.6.1 (R Core Team 2019) using a combination of published R packages: SpiecEasi for creation of microbial networks (version 1.1.1), the spinglass algorithm of the igraph package for creating microbial clusters (version 1.2.6), piecewiseSEM (version 2.1.0) in combination with nlme for structural equation modeling (version 3.1-152), the vegan package for plant and microbial community compositional data analysis (version 2.5-7), nls2 for calculating non-linear trends (version 0.3-3), QuantPsyc for extraction beta slopes from linear models and the stats (1.6), nlme and lme4 packages for linear models and linear mixed effects models including random effects (version 3.1-152 and 1.1-32, respectively). All packages and statistical methodologies are referenced in the Method and Supplementary Method sections. All R code is available via https://github.com/dintzandt/scripts_stability_and_soil_microbial_networks

For manuscripts utilizing custom algorithms or software that are central to the research but not yet described in published literature, software must be made available to editors and reviewers. We strongly encourage code deposition in a community repository (e.g. GitHub). See the Nature Portfolio guidelines for submitting code & software for further information.

Data

Policy information about availability of data

All manuscripts must include a data availability statement. This statement should provide the following information, where applicable:

- Accession codes, unique identifiers, or web links for publicly available datasets
- A description of any restrictions on data availability
- For clinical datasets or third party data, please ensure that the statement adheres to our policy

All raw data generated in this study have been deposited in the Zenodo digital data repository (https://doi.org/10.5281/zenodo.6695065). The raw microbial sequencing data generated in this study have been deposited in the NCBI SRA database under BioProject ID PRJNA931221 (https://www.ncbi.nlm.nih.gov/bioproject/931221). Fungal trait data is publicly available via the FungalTrait database (Põlme et al. 2020. Fungal Divers 105, 1–16). Czech Ellenberg values for flora are publicly available via Chytrý et al (2018. Preslia 90, 83–103). Source data are provided with this paper.

Research involving human participants, their data, or biological material

Policy information about studies with human participants or human data. See also policy information about sex, gender (identity/presentation), and sexual orientation and race, ethnicity and racism.

Reporting on sex and gender	No human research participants were used
Reporting on race, ethnicity, or other socially relevant groupings	No human research participants were used
Population characteristics	No human research participants were used
Recruitment	No human research participants were used
Ethics oversight	No human research participants were used

Note that full information on the approval of the study protocol must also be provided in the manuscript.

Field-specific reporting

Please select the one below that is the best fit for your research. If you are not sure, read the appropriate sections before making your selection.

☐ Life sciences ☐ Behavioural & social sciences ☒ Ecological, evolutionary & environmental sciences

For a reference copy of the document with all sections, see nature.com/documents/nr-reporting-summary-flat.pdf

Ecological, evolutionary & environmental sciences study design

All studies must disclose on these points even when the disclosure is negative.

Study description	In 2007, dry grassland communities of 44 perennial species were established in outdoor mesocosms on soil collected from a natural dry grassland site and from a site abandoned from arable agriculture 60 years before soil collection (30 plant communities each). Plant communities were left to establish until 2012 and no invasion of plant species from outside the sown species pool was allowed in this period. From 2012 onwards, plant species from outside the sown species pool invaded into the plant communities. Every year in the growing season, we determined the biomass proportion of each plant species in each community. After the growing season in 2019, we took soil cores to determine soil microbial biomass pools, microbial community composition and soil chemistry (6 soil cores from each community). We tested how plant community stability to plant species invasion was associated with microbial soil networks and soil chemical composition.
Research sample	Species-rich, dry grassland communities as well as soil prokaryotes and fungal communities. Dry grassland communities are biodiverse ecosystems, but these systems have been lost due to agricultural expansion. Restoring former agricultural land to dry grassland communities is challenging due to the success of invading, ruderal plant species that often take over these communities. In dry grassland communities established on natural grassland and abandoned arable soil and exposed to invasion of ruderal plant species, we therefore connected aboveground processes with belowground prokaryote and fungal communities.
Sampling strategy	In total 60 plant communities were established, 30 on natural grassland soil and 30 on abandoned arable soil. This sample size was chosen to be able to monitor natural variation in plant community development (gradient instead of a strictly replicated design with multiple treatments) and is higher than the number of plant communities that is typically used to calculate soil microbial networks.<br>44 dry grassland plant species were sown. The number of species was obtained from the site where the natural dry grassland soil was collected and includes all species typically growing at such natural locations.<br>Soil was sampled by taking soil cores of 6 cm in diameter and 36 cm length were taken at six random positions in each plant community. The aboveground plant community was sampled by cutting the aboveground biomass at 3 cm above the soil in each mesocosm.
Data collection	Plant community aboveground biomass was harvested every July and September from 2007 until 2011. From 2012 onward,

aboveground biomass was harvested only once a year in July. These time points are similar to management practises at the natural grassland site. Aboveground biomass was cut off 3 cm above the soil and from 2007 until 2011, biomass was sorted per plant species, dried at 60℃ for at least 48 h, after which dry weight was determined. From 2012 onward, plant species biomass was estimated by determining the percentile abundance of each plant species per mesocosm and multiplying this by the total aboveground biomass cut at 3 cm height and dried at 60℃ for at least 48 h. In 2014 and 2015, aboveground biomass was cut, but no species proportions were determined. See also: Münzbergová, Z. Seed density significantly affects species richness and composition in experimental plant communities. PLoS ONE 7, (2012).

After the growing season in December 2019, soil cores of 6 cm in diameter and 36 cm length were taken at six random positions in each plant community. Aboveground plant parts were removed and soil of the six cores was thoroughly mixed by passing it through a 2 mm mesh. Roots that did not pass the mesh were collected, dried at 60℃ for at least 48 h after which dry weight was determined. Subsamples from the mixed soils were taken for soil chemical determination of total nitrogen (N), total and organic C, plant available NO3-, NH4+ and NO2-, K, P and pH. Furthermore, subsamples for analyses of total bacterial, fungal and arbuscular mycorrhizal fungi (AMF) biomass using PLFA and NLFA analyses were taken as well as microbiome community composition using 16S and ITS amplicon sequencing. Data collection was lead by Zuzana Münzbergová.

Timing and spatial scale	Plant community aboveground biomass was harvested every July and September from 2007 until 2011. From 2012 onward, aboveground biomass was harvested only once a year in July. These time points are similar to management practises at the natural grassland site. Soil cores were sampled after the growing season in December 2019 to be able to determine the effect of the plant communities onto soil and microbial properties (directional based on the shift in time).
Data exclusions	No data was excluded
Reproducibility	Experiments are fully reproducible based on the description in the method section. For all taken measurements, standard and well-establishes protocols were used. All are referenced in the Methods section.
Randomization	Microbial OTUs were assigned to network clusters based on their responses across the 30 plant communities (natural grassland and abandoned arable separately). We clustered similarly responding OTUs in each network using the Spin-glass algorithm of the igraph package. This approach clusters OTUs based on both positive and negative edges as well as their weight. Present and absent edges as well as positive and negative edges were given a similar importance, and unlimited spins (clusters) were provided. Clustering was compared against a null-model (randomised networks) to test for a significant organisational structure. The occurrence of significant correlations between all pairwise comparisons of prokaryote and fungal clusters was also tested against a null-model (randomised networks).
Blinding	Blinding was not possible during data collection of aboveground species proportions because differences in plant communities were evident. All other collection and laboratory procedures were performed blinded. All data analyses were blinded, because we did not have a predisposed idea on the exact relations between the plant community and microbial networks and properties.

Did the study involve field work? ☒ Yes ☐ No

Field work, collection and transport

Field conditions	Field soil was collected from two locations: a dry natural grassland soil (excavated near Encovany, Czech Republic; 50°31'44.6"N, 14° 15'12.6"E) and a soil on which dry natural grasslands was turned into agricultural land, extensively managed and abandoned 60 years before soil was collected (excavated near Institute of Botany, Czech Academy of Sciences; 50°0'7.11"N, 14°33'20.66"E). The two soils mainly differed in soil nutrient availability with the natural grassland soil being significantly lower in total N, organic C and plant available P and K than the abandoned arable soil.
Location	Location of the natural grassland soil: 50°31'44.6"N, 14°15'12.6"E. Location of the abandoned arable soil: 50°0'7.11"N, 14° 33'20.66"E.
Access & import/export	The natural dry grassland soil was collected from a regular study location of the Institute of Botany, Czech Academy of Sciences in agreement with local authorities and farmers. The land from which the abandoned arable soil was collected is owned by the Institute of Botany, Czech Academy of Sciences.
Disturbance	Disturbance was minimalised by replacing the excavated soil and leaving the aboveground plant community intact.

Reporting for specific materials, systems and methods

We require information from authors about some types of materials, experimental systems and methods used in many studies. Here, indicate whether each material, system or method listed is relevant to your study. If you are not sure if a list item applies to your research, read the appropriate section before selecting a response.

Materials & experimental systems

n/a	Involved in the study	
[x]	[ ]	Antibodies
[x]	[ ]	Eukaryotic cell lines
[x]	[ ]	Palaeontology and archaeology
[x]	[ ]	Animals and other organisms
[x]	[ ]	Clinical data
[x]	[ ]	Dual use research of concern
[ ]	[x]	Plants

Methods

n/a	Involved in the study	
[x]	[ ]	ChIP-seq
[x]	[ ]	Flow cytometry
[x]	[ ]	MRI-based neuroimaging

Dual use research of concern

Policy information about dual use research of concern

Hazards

Could the accidental, deliberate or reckless misuse of agents or technologies generated in the work, or the application of information presented in the manuscript, pose a threat to:

No	Yes	
[x]	[ ]	Public health
[x]	[ ]	National security
[x]	[ ]	Crops and/or livestock
[x]	[ ]	Ecosystems
[x]	[ ]	Any other significant area

Experiments of concern

Does the work involve any of these experiments of concern:

No	Yes	
[x]	[ ]	Demonstrate how to render a vaccine ineffective
[x]	[ ]	Confer resistance to therapeutically useful antibiotics or antiviral agents
[x]	[ ]	Enhance the virulence of a pathogen or render a nonpathogen virulent
[x]	[ ]	Increase transmissibility of a pathogen
[x]	[ ]	Alter the host range of a pathogen
[x]	[ ]	Enable evasion of diagnostic/detection modalities
[x]	[ ]	Enable the weaponization of a biological agent or toxin
[x]	[ ]	Any other potentially harmful combination of experiments and agents
