# Supplementary material for: Plant community stability is associated with a decoupling of prokaryote and fungal soil networks
Source: Nat Commun. 2023 Jun 22;14:3736. doi: 10.1038/s41467-023-39464-8 (PMC10287681; doi:10.1038/s41467-023-39464-8)
Supplement: Supplementary file 3 — Description of Additional Supplementary Files [file 41467_2023_39464_MOESM3_ESM.pdf]

Title: Supplementary Data 1
Description:
- Exact statistical values for Figures 1 – 4
- Exact statistical values for Supplementary Figures 1 – 2

Title: Supplementary Data 2
Description:
- Sequences of used primers for 16S and ITS amplicon sequencing
